# Interplay between gonadal hormones and postnatal overfeeding in defining sex-dependent differences in gut microbiota architecture

**DOI:** 10.18632/aging.104140

**Published:** 2020-10-27

**Authors:** Jose A. Santos-Marcos, Alexia Barroso, Oriol A. Rangel-Zuñiga, Cecilia Perdices-Lopez, Carmen Haro, Miguel A. Sanchez-Garrido, Helena Molina-Abril, Claes Ohlsson, Pablo Perez-Martinez, Matti Poutanen, Jose Lopez-Miranda, Francisco Perez-Jimenez, Manuel Tena-Sempere, Antonio Camargo

**Affiliations:** 1 Maimonides Biomedical Research Institute of Cordoba (IMIBIC), Cordoba, Spain; 2 Lipids and Atherosclerosis Research Unit, Internal Medicine Unit, Reina Sofia University Hospital, Cordoba, Spain; 3 Department of Medicine, University of Cordoba, Cordoba, Spain; 4 CIBER Fisiopatologia de la Obesidad y Nutricion (CIBEROBN), Instituto de Salud Carlos III, Madrid, Spain; 5 Department of Cell Biology, Physiology, and Immunology, University of Cordoba, Cordoba, Spain; 6 Institute for Sustainable Agriculture (IAS), Spanish National Research Council (CSIC), Cordoba, Spain; 7 Department of Applied Mathematics I, University of Seville, Seville, Spain; 8 Centre for Bone and Arthritis Research, Institute of Medicine, the Sahlgrenska Academy at University of Gothenburg, Gothenburg, Sweden; 9 Institute of Biomedicine, Research Centre for Integrative Physiology and Pharmacology, University of Turku, 20014 Turku, Finland

**Keywords:** gut microbiota, sex steroids, gender, metabolism, miRNAs

## Abstract

Aging is associated with a decline in sex hormones, variable between sexes, that has an impact on many different body systems and might contribute to age-related disease progression. We aimed to characterize the sex differences in gut microbiota, and to explore the impact of depletion of gonadal hormones, alone or combined with postnatal overfeeding, in rats. Many of the differences in the gut microbiota between sexes persisted after gonadectomy, but removal of gonadal hormones shaped several gut microbiota features towards a more deleterious profile, the effect being greater in females than in males, mainly when animals were concurrently overfed. Moreover, we identified several intestinal miRNAs as potential mediators of the impact of changes in gut microbiota on host organism physiology. Our study points out that gonadal hormones contribute to defining sex-dependent differences of gut microbiota, and discloses a potential role of gonadal hormones in shaping gut microbiota, as consequence of the interaction between sex and nutrition. Our data suggest that the changes in gut microbiota, observed in conditions of sex hormone decline, as those caused by ageing in men and menopause in women, might exert different effects on the host organism, which are putatively mediated by gut microbiota-intestinal miRNA cross-talk.

## INTRODUCTION

Aging is the largest risk factor for cardiovascular diseases (CVD) [1]. However, coronary heart disease usually starts in women 10 years later than in men, a difference that increases to 20 years for cardiac events such as myocardial infarction [2, 3]. It has been shown that sex steroid hormones play a key role in CVD susceptibility, but the differences in sex steroid profiles between elder men and women are smaller when compared to earlier in life [4]; for instance, sex steroid cardio-protection in women disappears after menopause [5]. Likewise, the decline in testosterone (T) seen in aging men is associated with a greater likelihood of CVD [6]. The mechanisms involved in the sex difference in CVD are not yet fully understood, but it is crucial to develop strategies and therapies aimed at reducing the incidence of CVD.

The gut microbiota has been shown to be involved in the development of CVD [7], suggesting a potential role in the dimorphism of their incidence, as gender, in addition to other factors, such as age, genetic make-up and nutritional habits, impacts on gut microbiota architecture [8–10]. In fact, in recent years there has been accumulating evidence suggesting that the differences in the intestinal microbiota according to gender may be associated with the sex differences observed in the development of autoimmune, metabolic and CV diseases [11, 12]. Moreover, diet and nutrition influence the host and the microbial metabolites [13], which might be associated with the onset of human pathologies [14]. In fact, the composition of the intestinal microbiota depends on the interactions between diet and the host’s gender, and the therapies used to restore the dysbiosis of the gut microbiota associated to disease should be gender-specific.

We have previously shown that the intestinal microbiota from post-menopausal women presents a higher *Firmicutes/Bacteroidetes* (*F/B*) ratio than men, and a lesser abundance of short chain fatty acids (SCFA)-producing bacteria compared with the intestinal microbiota from pre-menopausal women, highlighting the influence of estrogens on gut microbiota architecture [15]. Moreover, we have also shown the differences in the intestinal microbiota architecture between post-menopausal women and age-matched men, which may stem from the actual differences in sex hormone levels in elder men and women and/or may reflect the residual influence of the dramatic differences in sex steroid profiles in early life between the sexes, and which may have a persistent effect on gut microbiota over time [9]. Moreover, intestinal microbiota transplant experiments in germ-free mice have recently demonstrated that the sex of the recipient animal shapes the composition of the intestinal microbiota [10]. In addition, it has been shown that males have a less diverse gut microbiota than their female littermates, a difference which is minimized with the castration of males, showing the influence of androgens on gut microbiome composition [16]. In fact, it has been shown that sex steroid manipulation during periods of early development alters gut microbiota [17].

However, the gender contribution to the sex differences in the gut microbiota, independently of sex steroid hormones, is not well understood, and may contribute to explaining the differences between genders in the incidence of cardiometabolic diseases. This set of interrelated conditions includes CVD, such as coronary heart disease, as well as metabolic diseases, such as type 2 diabetes and obesity. In order to shed light on the sex differences in the gut microbiota and the contribution of gonadal hormones and obesity to such differences, we explored here the sex-specific architecture of gut microbiota in gonadal-intact and gonadectomized rats of both sexes, alone or in combination with postnatal overnutrition.

## RESULTS

### Sex differences in gut microbiota according to nutritional status

We first explored differences between gonadal-intact male and female rats. In these studies, we found a higher α-diversity of the bacterial community in gonadal-intact females than in males, as assessed by both Shannon and Observed OTUs indexes under normal feeding (NL-CD) or postnatal overfeeding (SL-HFD) conditions (Supplementary Figure 1A).

In terms of bacterial composition, NL-CD males were characterized by higher *Elusimicrobia*, *Cyanobacteria*, and *Verrucomicrobia* phyla, whereas females were characterized by higher *Euryarchaeota* and *TM7* phyla. In postnatal overfed rats (SL-HFD), differences in *Cyanobacteria*, *Euryarchaeota* and *TM7* remained between sexes, in addition to higher *Bacteroidetes* and *Spirochaetes* phyla in males and higher *Firmicutes* phyla in females (Figure 1A; Supplementary Figure 2A). Moreover, whereas no differences in the *F/B* ratio were observed between sexes in animals under normal feeding, we observed a higher ratio in females than in males subjected to postnatal overfeeding (Figure 2).

**Figure 1:**
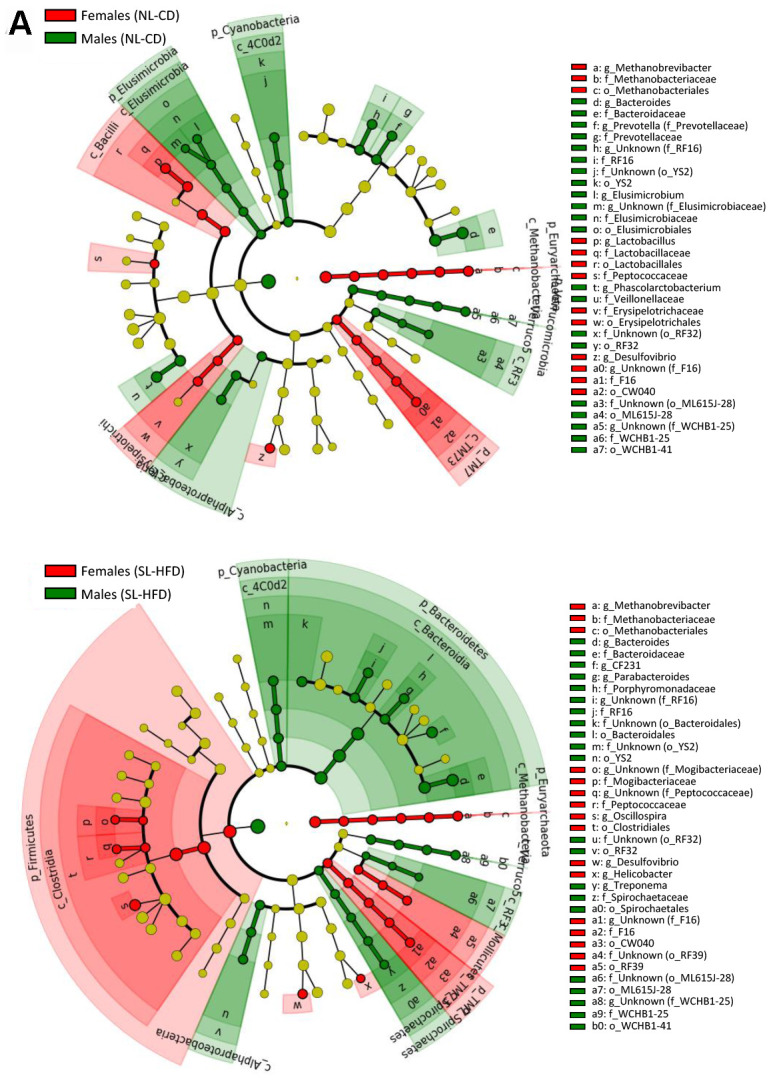
LEfSe analysis between sexes under normal feeding and overfeeding conditions in intact animals (**A**). Cladogram representing the taxonomic hierarchical structure of the identified differences between genders using Linear discriminant analysis effect size (LEfSe). Each filled circle represents one phylotype. Red denotes bacterial taxa statistically overrepresented in females; green denotes bacterial taxa overrepresented in males. Phylum and class are indicated by their names on the cladogram and the order, family, or genus are given in the key.

**Figure 1:**
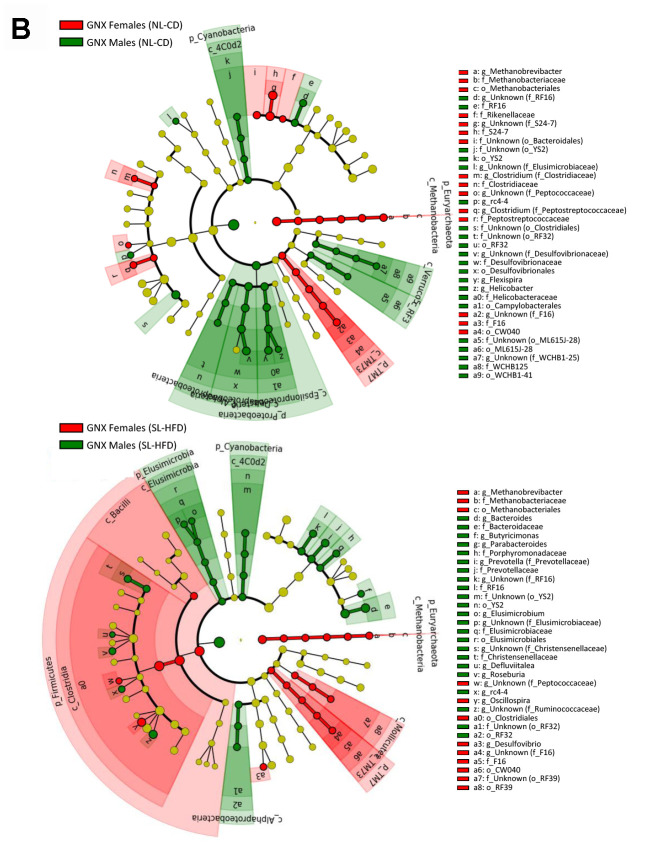
LEfSe analysis between sexes under normal feeding and overfeeding conditions in gonadectomized (**B**) animals. Cladogram representing the taxonomic hierarchical structure of the identified differences between genders using Linear discriminant analysis effect size (LEfSe). Each filled circle represents one phylotype. Red denotes bacterial taxa statistically overrepresented in females; green denotes bacterial taxa overrepresented in males. Phylum and class are indicated by their names on the cladogram and the order, family, or genus are given in the key.

**Figure 2:**
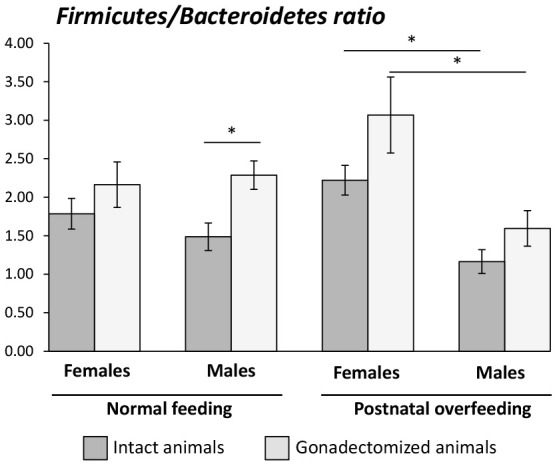
***Firmicutes/Bacteroidetes* ratio in intact and gonadectomized animals under normal feeding and overfeeding conditions.** ^*^P<0.05 in One-way ANOVA statistical analysis.

### Gut microbiota differences between sexes in gonadectomized animals

We next evaluated the differences between gonadectomized (GNX) males and females, under normal feeding or overfeeding conditions.

We found no differences in the α-diversity of the bacterial community between GNX males and females, regardless of the feeding condition (Supplementary Figure 1A). In terms of bacterial composition, most of the differences at phylum level found between male and female intact rats, both under normal feeding and postnatal overfeeding conditions, were also presented in GNX animals. In fact, differences in *TM7*, *Cyanobacteria*, and *Euryarchaeota* phyla under normal feeding remained, whereas differences in *Elusimicrobia* and *Verrucomicrobia* phyla were absent. In addition, GNX males had higher *Proteobacteria* than GNX females. In postnatal overfeeding condition, differences in *Cyanobacteria, Euryarchaeota*, *Firmicutes* and *TM7* remained between GNX males and females, whereas differences in *Bacteroidetes* and *Spirochaetes* disappeared. Additionally, GNX males had higher *Elusimicrobia* than GNX females (Figure 1B; Supplementary Figure 2B). Moreover, whereas no differences in the *F/B* ratio were observed between sexes in GNX animals under normal feeding, we detected a higher *F/B* ratio in GNX females than in GNX males following postnatal overnutrition (Figure 2). In addition, from 55 bacterial genera included in LEfSe analysis, the abundance of 11 of these was different between sexes under normal feeding conditions, and the difference in the abundance of 6 of these disappeared after gonadectomy. Moreover, 8 additional bacterial genera were differentially represented between sexes only after gonadectomy in conditions of normal feeding. By contrast, the abundance of 13 genera was different between sexes under postnatal overfeeding condition, and the difference in the abundance of 5 of these disappeared after gonadectomy. In addition, 9 additional bacterial genera were differentially represented between sexes only after gonadectomy in overfed animals (Supplementary Table 1).

### Impact of postnatal overfeeding in sex steroid hormones levels

Next, we evaluated the relationship between the obesogenic insult (postnatal overfeeding) and circulating sex steroids by measuring their plasma levels using the sensitive gas chromatography-tandem mass spectrometry method.

We found lower T, dihydrotestosterone, and androstenedione serum levels in males subjected to postnatal overfeeding that in those with normal feeding. No differences in the serum concentrations of these hormones, whose levels were much lower than in males, were found in females, regardless of their nutritional status. However, we found lower serum levels of estradiol (E_2_) in females under normal feeding than in those subjected to postnatal overfeeding, whereas no differences in progesterone or estrone (E_1_) levels (the latter was only detected in females) were found (Table 1). Because of the surgical removal of the gonads, sex steroid levels were not assessed in GNX male or female rats.

**Table 1:** Sex steroid plasma levels in intact animals under normal feeding and postnatal overfeeding conditions.

		***Males***				***Females***		
***Sex steroid (pg/ml)***		***NL-CD***	***SL-HFD***	***p-value***		***NL-CD***	***SL-HFD***	***p-value***
*Testosterone*		9347.12±2850.11	2070.24±421.49	0.027		80.49±19.95	122.16±36.97	0.323
*Dihydrotestosterone*		87.23±22.91	22.63±5.31	0.016		3.74±1.05	8.15±2.33	0.097
*Androstenedione*		669.91±169.78	133.64±20.40	0.007		47.47±2.94	73.38±14.72	0.089
*Estradiol*		0.32±0.32	n.d.	n.a.		3.19±0.13	8.46±2.33	0.032
*Progesterone*		909.23±125.72	765.00±317.08	0.679		18380.32±2037.92	16474.30±3361.90	0.626
*Estrone*		n.d.	n.d.	n.a.		1.00±0.27	2.79±1.68	0.278

NL-CD: normal litter, control diet. SL-HFD: small litter, high fat diet. Plasma was collected at PND-150 for determination of sex steroids by mass spectrometry. p-value: One-way ANOVA statistical analysis. n.d.: not detectable; n.a.: not available.

### Sex-dependent metabolic disruption after gonadectomy

Further, we studied the sex-dependent metabolic alterations, alone or in combination with postnatal overnutrition, caused by GNX in males and females.

No differences in body weight (BW) were found between gonadal-intact and GNX males. By contrast, the BW of GNX females was higher than in intact females. We also observed in females an increase in plasma leptin levels in parallel with changes in BW after gonadectomy (Table 2).

**Table 2:** Metabolic parameters in intact and gonadectomized animals under normal feeding and under postnatal overfeeding.

		***Males***				***Females***		
		***non-GNX***	***GNX***	***p-value***		***non-GNX***	***GNX***	***p-value***
*Body weight (g)*	***NL-CD***	356.96±12.47	328.78±7.71	**0.079**		223.53±5.62	256.31±5.45	**0.006**
	***SL-HFD***	478.61±7.65	481.31±15.16	**0.826**		260.30±8.65	319.73±8.92	**<0.001**
	***p-value***	**<0.001**	**<0.001**			**0.011**	**<0.001**	
*Leptin (ng/ml)*	***NL-CD***	10.26±2.90	8.69±2.44	**0.691**		3.77±0.51	7.43±0.95	**0.014**
	***SL-HFD***	34.03±5.02	32.45±3.79	**0.813**		10.19±1.81	22.62±4.09	**0.010**
	***p-value***	**0.005**	**0.001**			**0.014**	**0.005**	
*AUC GTT*	***NL-CD***	18230.00±818.40	17381.43±1473.46	**0.404**		21231.25±1434.01	20641.25±1452.55	**0.601**
	***SL-HFD***	23947.50±1215.50	24797.50±1255.93	**0.558**		22875.00±1000.87	24111.25±1329.62	**0.514**
	***p-value***	**0.001**	**0.005**			**0.330**	**0.086**	
*Δ AUC GTT*	***NL-CD***	4835.00±531.22	3855.71±758.97	**0.118**		6126.25±769.10	7021.25±625.65	**0.348**
	***SL-HFD***	3798.75±1084.42	8687.50±1328.90	**0.023**		8340.00±1237.97	6066.25±542.50	**0.089**
	***p-value***	**0.394**	**0.003**			**0.078**	**0.230**	
*AUC ITT*	***NL-CD***	5986.25±240.34	7126.25±585.95	**0.130**		6593.75±215.38	5890.00±264.53	**0.111**
	***SL-HFD***	7705.00±268.30	7623.75±327.63	**0.841**		6275.00±138.07	7401.25±553.07	**0.132**
	***p-value***	**0.005**	**0.561**			**0.327**	**0.030**	

NL-CD: normal litter, control diet. SL-HFD: small litter, high fat diet. GNX: gonadectomized animals. Non-GNX: intact animals. Glucose tolerance test (GTT) was performed at PND-120. Insulin tolerance test (ITT) was performed one week later than GTT. Body weight corresponds to PND-150 animals. p-value: One-way ANOVA statistical analysis.

Glucose tolerance, as measured by the area under the curve (AUC) of glucose during glucose tolerance test (GTT), was significantly worse in males subjected to postnatal overfeeding (SL-HFD), in both gonadal-intact and GNX conditions. However, GNX per se did not alter AUC GTT values in any of the two nutritional conditions. Nonetheless, we observed a higher ΔAUC GTT (as a net increment of the AUC over basal levels) of glucose in GNX males vs. intact males that were raised under postnatal overfeeding conditions. In turn, in gonadal-intact female rats, AUC GTT was not altered by SL-HFD, while in GNX females, the same obesogenic diet tended to increase glucose intolerance, although this change did not reach statistical significance (P=0.086). Regarding insulin sensitivity, obese (SL-HFD) males displayed insulin resistance, defined by significantly higher AUC during the insulin tolerance test (ITT) values than in lean (NL-CD) males, but GNX did not worsen insulin sensitivity neither in NL-CD or SL-HFD conditions. In contrast, higher insulin resistance was detected in SL-HFD females only when they were previously ovariectomized (Table 2).

### Gonadal hormone contribution to gut microbiota structure

Next, we evaluated the hormonal contribution to gut microbiota structure by comparing the gut microbiota of intact versus GNX males and intact versus GNX females.

We found a higher α-diversity of the bacterial community in GNX males than in intact males as assessed by both Shannon and Observed OTUs indexes under normal feeding conditions, while no differences were found in overfed males (a trend for higher α-diversity in GNX males was observed). However, ovariectomy in females did not change these diversity indexes (Supplementary Figure 1B).

In terms of bacterial composition under normal feeding conditions, we observed that the gut microbiota of GNX males was characterized by higher *Firmicutes*, *Deferribacteres* and *TM7* phyla, and lower *Bacteroidetes* phylum, compared with intact males. By contrast, in animals subjected to postnatal overfeeding, the gut microbiota from GNX and intact males differed in the minority phylum *Elusimicrobia*, which was more abundant in GNX males. On the other hand, gonadectomy slightly impacted on the gut microbiota from females under normal feeding, with lower *Proteobacteria* phylum in GNX females, whereas in conditions of postnatal overfeeding, the gut microbiota from GNX females was characterized by higher *Elusimicrobia* and *Spirochaetes* phyla and lower *Actinobacteria* phylum (Figure 3; Supplementary Figure 3).

**Figure 3:**
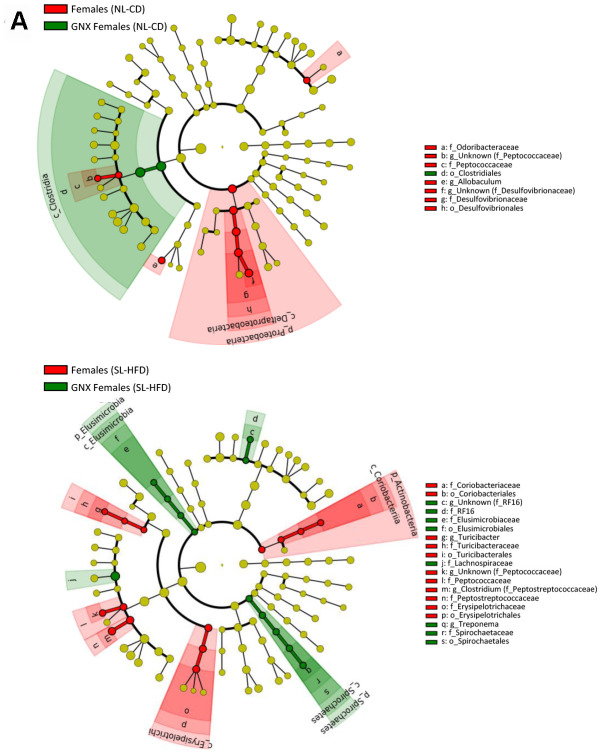
LEfSe analysis between intact and gonadectomized animals under normal feeding and overfeeding conditions in females (**A**). Cladogram representing the taxonomic hierarchical structure of the identified differences between genders using Linear discriminant analysis effect size (LEfSe). Each filled circle represents one phylotype. Red denotes bacterial taxa statistically overrepresented in intact animals; green denotes bacterial taxa overrepresented in gonadectomized animals. Phylum and class are indicated by their names on the cladogram and the order, family, or genus are given in the key.

**Figure 3:**
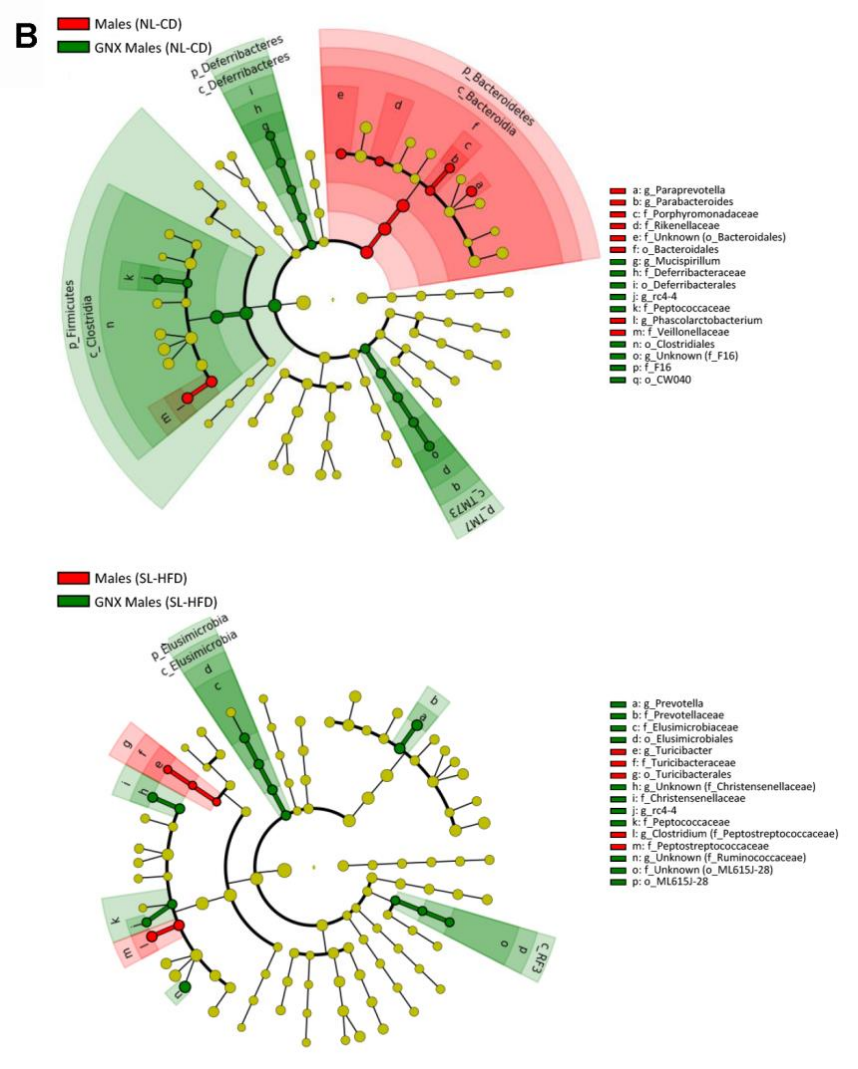
LEfSe analysis between intact and gonadectomized animals under normal feeding and overfeeding conditions in males (**B**). Cladogram representing the taxonomic hierarchical structure of the identified differences between genders using Linear discriminant analysis effect size (LEfSe). Each filled circle represents one phylotype. Red denotes bacterial taxa statistically overrepresented in intact animals; green denotes bacterial taxa overrepresented in gonadectomized animals. Phylum and class are indicated by their names on the cladogram and the order, family, or genus are given in the key.

### Microbiota putatively modulates host metabolism via miRNAs

Finally, we evaluated the potential role of miRNAs on the dialogue (cross-talk) between gut microbiota and host organism in response to changes in sex hormones and nutritional status.

**Figure 4:**
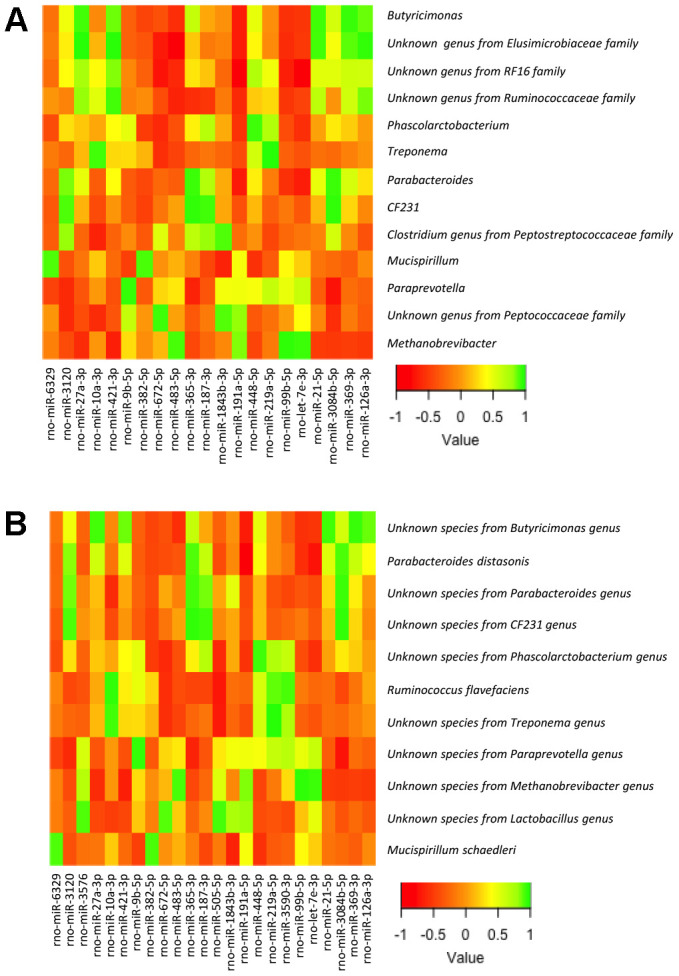
Heatmap from the Pearson’s correlation coefficient between the bacterial genera (**A**) and species (**B**) identified by LEfSe analysis and the expression levels of the miRNAs in the large intestine.

**Figure 5:**
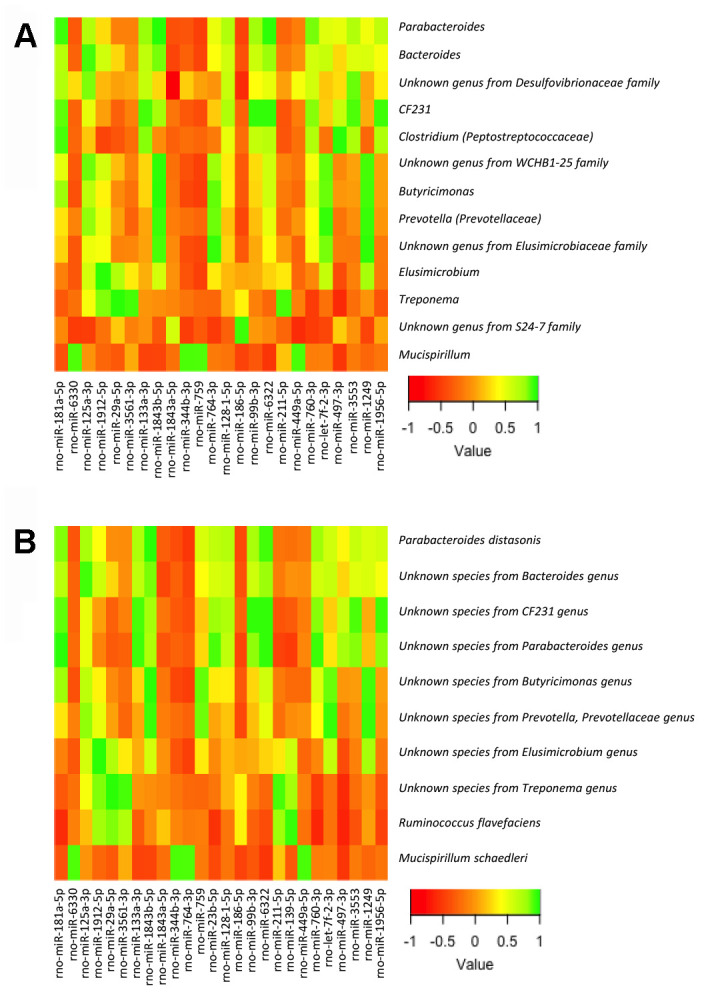
Heatmap from the Pearson’s correlation coefficient between the bacterial genera (**A**) and species (**B**) identified by LEfSe analysis and the expression levels of the miRNAs in the small intestine.

To this end, we analyzed the relationship between the bacterial taxa identified by LEfSe analysis according to gender, sex hormones and obesity, and the expression levels of the miRNAs in the small and large intestine, determined by expression microarray analysis. Of note, we did not include in the analysis all the bacterial taxa but only those identified by LEfSe analysis in order to reduce random associations (Supplementary Tables 2–5; Figures 4, 5). From 758 miRNAs tested, the expression of 99 and 101 miRNAs was detectable in the large and small intestine, respectively, in at least 7 of the 8 experimental groups. From these, 54 miRNAs were detectable in both the large and small intestine. From the correlation analysis, we selected 27 miRNAs in the small intestine and 25 in the large intestine (1 miRNA were shared by both the large and small intestine), in which Pearson’s correlation coefficient was > 0.9 or < -0.9 and P<0.01. Further, we performed a supplemental analysis with the 51 selected miRNAs using the DIANAtools V.3. DIANA-miRPath is a web-server which provides accurate statistics and can accommodate advanced pipelines. miRPath can utilize predicted miRNA targets (in CDS or 3’-UTR regions) provided by the DIANA-microT-CDS algorithm or even experimentally validated miRNA interactions derived from DIANA-TarBase [18]. Thus, in addition to several KEGG pathways related with metabolism, our approach detected miRNA-mediated associations between the gut microbiota and sex steroid-related pathways. The functions of KEGG, in which selected miRNAs were assigned, included the metabolism of lipids, amino acids, cofactors and vitamins, signal transduction, and endocrine systems. Specifically, insulin, GnRH, estrogen, and prolactin signaling pathways, as well as progesterone-mediated oocyte maturation, were involved (Supplementary Tables 6, 7).

## DISCUSSION

Our study documents that many of the differences in the gut microbiota found between males and females, both under normal and overfeeding conditions, persisted after gonadectomy. However, removing the sex hormones shaped several gut microbiota features towards a more deleterious profile, especially in females, mainly when animals were subjected to postnatal overfeeding. In addition, our study also shows that overnutrition in females significantly increased *F/B* ratio as compared with males.

Previous observations in humans showed that the *F/B* ratio, which is of major importance in the development of obesity as it increases in this condition [19], is higher in women than in men under obesity conditions [9], and increases in women after menopause [15]. Consistent with this, our study showed that the *F/B* ratio was higher in females than in males subjected to postnatal overfeeding, both in intact and GNX animals, even taking into account that the gonadectomy of males, as previously shown in mice [20], increased the *F/B* ratio. In line with this, our study showed that this increase was proportional to the prevailing T levels, being higher in normal fed animals than in postnatal overfed males, which showed a decline of endogenous T levels due to obesity [21].

By contrast to males, in which no changes in BW were observed after gonadectomy, in females, ovariectomy caused an increase in BW in parallel with the rise in leptin levels. This observation may be explained on the basis of the anti-obesity effect of estrogens through decreasing food intake and increasing energy expenditure [22]. In fact, animal studies have shown that while females are relatively resistant to diet-induced obesity, ovariectomy reverses this protective effect [23], whereas estrogens protect ovariectomized females from obesity [24].

It has been proposed that the gender differences in the incidence during adulthood of cardiometabolic diseases - a set of interrelated cardiovascular and metabolic diseases - may be explained, at least partially, by sex-specific effects of dietary factors during early stages of life, in addition to maternal conditions in the uterus [25]. Herein, we show that the postnatal overfeeding (continued with an obesogenic diet after weaning) of females had a discernible impact on the *F/B* ratio, a phenomenon that was not observed in males. This contrasts with previous observations from studies in animal models, mostly performed only in males, that showed an obese microbiota pattern characterized by a high *F/B* ratio [19]. Thus, our study suggests that persistent overnutrition since lactation may have a durable influence on the sensitivity of gut microbiota to diet-induced changes in the adulthood. This idea is supported by the fact that obesity in childhood, which is associated with a higher risk of obesity in adulthood [26], is linked to alterations in gut microbiota at an early age [27]. Moreover, the influence of postnatal overfeeding in shaping gut microbiota in females, but not in males, may also help to explain the inconsistent results surrounding changes in *F/B* ratio in several studies in humans, as the period of life in which overfeeding triggered obesity seems to be important for determining gut microbiota dysbiosis. In fact, while several studies have shown an increased *F/B* ratio in obesity [28, 29], others did not confirm these observations [30], or even showed that this ratio was reduced in obese subjects [31].

Moreover, postnatal overfeeding and gonadectomy also impacted differentially on several bacterial taxa at lower hierarchical levels. In relation to metabolic disease, our study showed that the lower abundance of *Bacteroides* genus and *Prevotellaceae* family in females as compared with males, which has been associated to metabolic syndrome in humans [32], disappeared after gonadectomy under normal feeding conditions, but not under postnatal overfeeding conditions. In addition, we also observed a higher abundance of *Clostridiaceae* family in females after gonadectomy under normal feeding conditions; this family is also related with metabolic syndrome in humans [33].

All together, these alterations in gut microbiota suggest a higher impact of GNX in females when animals were postnatally overfed, a phenomenon which is consistent with previous observations in humans, in which the differences in the gut microbiota between men and postmenopausal women are influenced by the grade of obesity [9, 15]. In addition, the combination of both overfeeding and sex steroid removal by gonadectomy seems to have a more deleterious effect in females than in males, as suggested by the abundance of two SCFA-producing bacterial genera, *Butyricimonas* and *Roseburia*. In fact, the lower abundance of these bacterial genera in GNX females under postnatal overfeeding supports the idea that the microbiota in males, but presumably not in females, is able to adapt itself when it is exposed to high caloric supply early on life, and is able to maintain a higher SCFA production than in females. This, therefore, may impact differentially on disease predisposition between genders, and might also affect disease incidence. In fact, it has been described that metabolic diseases increase after menopause in women in parallel with estrogen depletion [5], which is also related with gender differences in fat distribution [34].

We also explored whether the dialogue, or cross-talk, between gut microbiota and host organism in response to changes in sex hormones and nutritional status can take place through regulation of miRNA expression in the small and large intestines, which is increasingly recognized as transmitters or decoders of dysbiosis into cardiometabolic diseases [35, 36]. Based on KEGG pathways, our study identified miR-23b-5p and miR-186-5p, expressed in the small intestine, as potential modulators of steroid biosynthesis, in response to gut microbiota changes. In fact, we found a relationship in terms of abundance-expression of these miRNAs with an unknown bacterial species from the *Parabacteroides* genus (in the case of miR-23b-5p) and with an unknown genus from the *S24-7* family (miR-186-5p). These findings point out that these bacterial taxa might be related in modulating steroid biosynthesis. In addition, the expression in small intestine of another two miRNAs, miR-181a-5p and miR-139-5p, both involved in the estrogen signaling pathway, was related with the intestinal abundance of *Parabacteroides* and *Clostridium* (from *Peptostreptococcaceae* family) in the first case, and with *Ruminococcus flavefaciens* in the second.

We also identified platelet activation as one of the pathways that may be modulated by gut microbiota-miRNAs cross-talk in response to sex steroid-related alterations. In fact, it was recently shown that T reduces platelet activation in elderly people [37]. Taking into account the decline in T seen in aging [6], a potential role of the gut microbiota through miRNA actions inducing changes in blood platelets might be suggested. This idea is also supported by the previously described aging-induced changes in the gut microbiota [38].

Moreover, our study showed that gut microbiota-miRNAs cross-talk may also influence the intestinal barrier integrity through modulation of adherens junctions, which, together with the tight junction, provide important adhesive contacts between epithelial cells, and are involved in intestinal barrier permeability [39]. However, this potential mechanism would be complementary to the direct effect through bacterial species involved in the stability of the mucosal layer [40]. In addition, diet may also exert its effect through the cross-talk between gut microbiota and the intestinal expression of miRNAs, as evidenced by the relationship between miR-125a-3p, involved in adherens junctions, and the abundance of *Bacteroides* in the small intestine, associated to a meat-rich diet, as most of the species are bile acid resistant [41].

Intestinal absorption of bacterial components, such as endotoxin lipopolysaccharide, induces inflammation through toll-like receptor activation, which may promote insulin resistance [42]. In line with this, our study also showed the relationship between the intestinal expression of the insulin signaling-related miR-27a-3p, and the abundance of *Butyricimonas* in the large intestine. Moreover, this bacterial genus is a butyrate-producer [43], which may also be involved in insulin signaling, as SCFA increases the action and release of insulin through intestinal incretins [44, 45]. Furthermore, SCFA are also involved in energy metabolism and appetite regulation [46], which may be partially responsible of the weight gain in females after GNX, a condition in which the abundance of this genus is higher in males than in females (GNX, SL-HFD). Additionally, miR-27a-3p is also involved in mediating sex-steroid actions in other tissues, such as progesterone-mediated oocyte maturation, therefore supporting the view that the cross-talk between gut microbiota and the host via specific miRNAs may also involve gonadal steroid mediated events. This idea is also supported by the relationship found between miR-181a-5p, related with the estrogen signaling pathway, and the abundance of *Parabacteroides* in the small intestine, a genus associated to sulphate assimilation but also a producer of SCFA [47]. Overall, our results support the idea that gut microbiota-miRNA cross-talk may serve as decoder of changes in the gut microbiota composition into the host metabolism, in line with previous data [35, 36].

**Figure 6:**
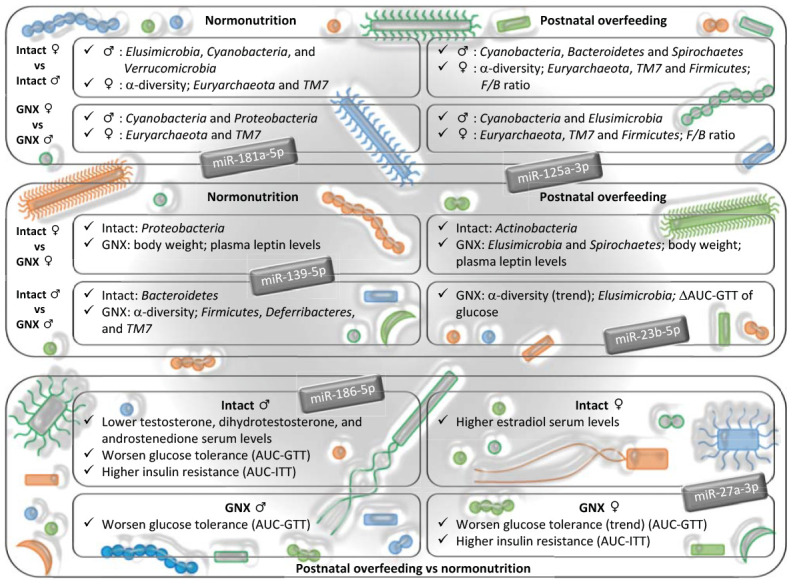
**Impact of gonadal hormone depletion, alone or combined with postnatal overfeeding, on the sex-differences in gut microbiota, subsequent metabolic alterations and potential miRNAs involved.** Upper panel: gender differences in intact and gonadectomized animals. GNX, gonadectomized animals. The bacterial taxa indicated are more abundant in the gender shown by the symbol. Intermediate panel: impact of depletion of gonadal hormones. The variables indicated are more abundant in the animal model shown (intact or GNX animals). ΔAUC, delta area under the curve. GTT, glucose tolerance test. Lower panel: effect of postnatal overfeeding on intact and gonadectomized animals (in this panel, text refers to effect found in postnatal overfeeding as compared with normonutrition). ITT, insulin tolerance test. miRNAs shown are putatively involved in the dialogue between gut microbiota and host organism in response to changes in sex hormones and nutritional status, and related with the insulin signaling pathway, steroid biosynthesis, the estrogen signaling pathway, adherens junctions and progesterone-mediated oocyte maturation.

In conclusion, our study documents the contribution of gonadal hormones to defining sex-dependent differences on gut microbiota, and discloses a potential role of gonadal hormones in shaping gut microbiota, as consequence of the interaction between sex and nutrition (Figure 6). Thus, the development of therapies aimed at restoring gut microbiota alterations in elderly people, in order to reduce the risk of diseases such as CVD, should be gender-specific. Our data suggest that the changes in gut microbiota, observed in conditions of sex hormone decline, such as those caused by ageing in men and menopause in women, may exert different effects on the host organism, which are putatively mediated by gut microbiota-miRNA cross-talk.

## MATERIALS AND METHODS

### Animals and diets

Wistar male and female rats bred in the vivarium of the University of Cordoba were used. The animals were maintained at 22 ± 1°C under constant conditions of light (14 hours) with free access to water. The experimental animals were fed a control diet (CD), D12450B (10%, 20%, and 70% calories from fat, protein, and carbohydrate, respectively), or a high-fat diet (HFD), D12451 (45%, 20%, and 35% calories from fat, protein and carbohydrate, respectively; Research Diets Inc., New Brunswick, NJ, USA). All the experimental protocols were approved by Cordoba University Ethical Committee for animal experimentation and conducted in accordance with the European Union guidelines for use of experimental animals.

### Experimental design

On postnatal day (PND)-1, pups were cross-fostered and reared in two different litter sizes: small litters (SLs) (4 pups per litter; as a model of postnatal overnutrition) or normal litters (NLs) (12 pups per litter), as extensively described previously [48–50]. The animals were weaned at PND-23 and housed in groups of four or five rats per cage. From weaning onwards, subgroups of NL and SL females and males were fed CD or HFD *ad libitum*, respectively; thus, two experimental groups (NL-CD and SL-HFD) were generated, representative of the lean and obese phenotype, respectively. On PND-90, subsets of animals from each group were subjected to gonadectomy, via bilateral abdominal approach in the case of females, or via scrotal route in case of males, as a model of cessation of gonadal secretions. At PND-120, the animals were subjected to a GTT, and one week later to an ITT to assess the development of insulin resistance in the different experimental models.

Experiments were terminated at PND-150, both in gonadal-intact and GNX animals; the latter, 60 days after surgical removal of the gonads. At this stage, phenotypic indices and serum biochemical/hormonal parameters were monitored; sampling in the groups of intact females was carried out at the same stage of the ovarian cycle, namely, diestrus-1. Rats were euthanized by decapitation and trunk blood was collected for analyses. Additionally, sections of small and large intestine were dissected and fecal samples were obtained from the different study groups directly from stool expulsion stimulated by manual handling. Samples were frozen in liquid nitrogen and stored at -80 °C until analysis.

### Phenotypic indices and hormonal measurements

Terminal BW was monitored on PND-150 intact and GNX rats. Glucose concentrations were measured in blood samples, taken from the experimental animals at PND-120 after overnight fasting. In PND-150, serum levels of leptin were assayed by double-antibody RIA, using the kit provided by EMD MILLIPORE (St. Charles, MO, USA). The sensitivity limit of the assay was 0.8 ng/mL, and the intra- and inter-assay coefficients of variation were less than 4% and 9%, respectively. In addition, in intact animals of both experimental groups (NL-CD and SL-HFD), sex steroid plasma levels were determined using a thoroughly validated, sensitive gas chromatography-tandem mass spectrometry method, in keeping with previous references [51, 52]. Next, the serum levels of T, dihydrotestosterone, androstenedione, progesterone, E_1_ and E_2_ were measured. The lowest levels of quantification in the assay were: 8 pg/mL for T, 2.5 pg/mL for dihydrotestosterone, 12 pg/mL for androstenedione, 74 pg/mL for progesterone, and 0.5 pg/mL for E_1_ and E_2_, in line with previous references [51, 52].

### Glucose tolerance tests and insulin tolerance tests

To assess glucose handling in all the experimental groups, the rats were subjected to GTT on approximately PND-120. The rats were fasted overnight and subsequently received an intraperitoneal (ip) bolus of glucose (1 g/kg BW). Blood glucose levels were determined before (0) and at 20, 60, and 120 minutes post administration. After complete recovery, one week later, insulin sensitivity was assessed using ITT. For this, the rats were fasted overnight, followed by an ip injection of 1UI insulin (Sigma-Aldrich, St. Louis, MO) per kg BW. Blood glucose levels were measured before (0) and at 20, 60, and 120 minutes after insulin administration. Integral glucose changes and net increases in integral glucose levels were estimated as area under the curve (AUC) and delta area under the curve (ΔAUC), respectively, during the 120 min period after the glucose or insulin administration, as calculated by the trapezoidal method. All glucose concentrations were measured using a handheld glucometer (ACCU-CHECK Aviva; Roche Diagnostics).

### Intestinal microbiota analysis

DNA extraction from feces was performed using the QIAamp DNAStool Mini Kit Handbook (QIAGEN, Hilden, Germany), following the manufacturer’s instructions. The microbiota composition analysis of the fecal samples was performed on a MiSeq Illumina platform (Illumina, San Diego, CA, USA), according to the manufacturer's instructions. Briefly, polymerase chain reaction (PCR) was performed using 0.2 μM of the primer 5’-TCGTCGGCAGCGTCAGATGTGTATAAGAGACAG-3' and 5'-GTCTCGTGGGCTCGGAGATGTGTATAAGAGACAG-3’ [53] to generate amplicons containing the hypervariable region V3 of the 16s rRNA gene. KAPA HiFi HotStart ReadyMix (KAPABIOSYSTEMS) and 1.25 μl of extracted DNA (5 ng/μl in 10 mM Tris pH8.5) were used with the following PCR parameters: 3 minutes denaturation at 95°C, followed by 25 cycles (30 s at 95°C, 30 s at 60°C, 30 s at 72°C) and a final extension at 72°C for 5 min. The amplicon purification was performed using Agentcourt AMPure XP beads (Beckman Coulter). A second PCR reaction attaches dual indices and Illumina sequencing adapters. For this, the Nextera XT Index Kit was used. This PCR was performed with a KAPA HiFi HotStart ReadyMix (KAPABIOSYSTEMS), 5 μl of the previous amplicon, 5 μl of each Nextera XT Index Primer 1(N7xx) and 5 uL of each Nextera XT Index Primer 2(S5xx), with the following cycle parameters: 3 minutes denaturation at 95°C, followed by 8 cycles (30 s at 95°C, 30 s at 55°C, 30 s at 72°C), and a final extension at 72°C for 5 min. The PCR product purification was performed using Agentcourt AMPure XP beads (Beckman Coulter). Sequencing data were analyzed and visualised using QIIME 2 v. 2019.7 [54]. Demultiplexed single-end reads containing V3 hypervariable region were truncated at 212 bp (Quality score median >30), and denoised using the DADA2 method [55]. After filtering, the high-quality reads of the 64 samples (n = 8 for each group) ranging from 224,029 to 18,682 sequence counts were taken, with the rarefaction depth established at 18,500 sequence counts. Bacterial α-diversity across the samples was calculated using the observed OTUs and Shannon indexes [56]. Principal component analysis of community structure (beta-diversity) was performed using the unweighted and weighted UniFrac distance metrics [57] and analyzed by permutational multivariate analysis of variance (PERMANOVA). Taxonomy was assigned to the high-quality reads using q2-feature-classifier [58] with a sequence identity threshold of 99%, interrogating the sequences with the Greengenes database (13_8) [59]. To be consistent with the taxonomic data obtained from 16S rRNA, only taxa in the bacteria domain were included in the statistical analysis. The relative taxonomic abundance was measured as the proportion of reads over the total in each sample assigned to a given taxonomy. To exclude bacterial taxa that were not present in the majority of samples, a cut-off for exclusion was fixed; only bacterial taxa containing sequence reads in at least 75% of total samples were considered. Linear discriminant analysis (LDA) effect size (LEfSe) (http://huttenhower.sph.harvard.edu/galaxy/) was used to compare groups at baseline and visualize the results using taxonomic bar charts and cladograms [60].

### RNA isolation from the small and large intestine

Frozen tissue was ground to a fine powder in liquid nitrogen, using a mortar and pestle. RNA was isolated with the commercial kit Direct-zol^TM^ RNA MiniPrep Plus (Zymo Research Corp., CA, USA, and quantified using the v3.5.2 Nanodrop ND-1000 spectrophotometer (Nanodrop Technologies, Cambridge, UK).

### miRNA expression analysis

miRNA expression profiles were generated using the SurePrint Rat miRNA Microarrays (Rat miRNA 8x15K Microarray, Release 21.0, Agilent Technologies Inc., Santa Clara, CA, USA). RNA samples of each experimental group were pooled and labeled using the miRNA Labeling and Hyb Kit (Agilent Technologies Inc.), according to the manufacturer's instructions. Hybridization was performed using this latter kit, also according to the manufacturer's instructions. Microarray images of each slide were obtained with a Gene Pix 4000B scanner (Axon Instruments, Union City, CA, USA). Image quantization was performed using Agilent Feature Extraction Software (Agilent Technologies Inc.). Raw microarray data were analyzed using the limma R package [61]. Spots with foreground mean and median differing by more than 50 were filtered out and data quality was checked using limma tools. Background correction was performed using saddle-point approximation in the normal-exponential convolution method Normexp [62]. Next, within arrays Print-tip loess [63] and between arrays quantile were used for normalization. Finally, replicate spots in the array data were averaged.

### Software for miRNA analysis

To identify the role of selected miRNAs in the cellular processes, we performed an analysis using the DIANAtools V.3. DIANA-miRPath is a web-server (http://diana.imis.athena-innovation.gr/DianaTools/index.php), which provides accurate statistics and can accommodate advanced pipelines. DIANA-miRPath can utilize predicted miRNA targets (in CDS or 3’-UTR regions) provided by the DIANA-microT-CDS algorithm or even experimentally validated miRNA interactions derived from DIANA-TarBase [18].

### Statistical analysis

The PASW statistical software package, version 20.0 (IBM Inc., Chicago, IL, USA), was used for statistical analysis of the data. We used One-way ANOVA to test the differences in the plasma metabolites between groups of animals. Pearson’s correlation test was used to evaluate the relationship between miRNA intestinal expression and bacterial taxa abundance. Data are presented as mean ± standard error of the mean. P-values <0.05 were considered statistically significant in all the statistical analyses.

## Supplementary Material

Supplementary Figures

Supplementary Table 1

Supplementary Table 2

Supplementary Table 3

Supplementary Table 4

Supplementary Table 5

Supplementary Table 6

Supplementary Table 7
