# Supplementary material for: Interplay between gonadal hormones and postnatal overfeeding in defining sex-dependent differences in gut microbiota architecture
Source: Aging (Albany NY). 2020 Oct 27;12(20):19979–20000. doi: 10.18632/aging.104140 (PMC7655199; doi:10.18632/aging.104140)
Supplement: Supplementary Figures [file aging-12-104140-s001..pdf]

SUPPLEMENTARY FIGURES

A **Sex effect on alpha diversity.**

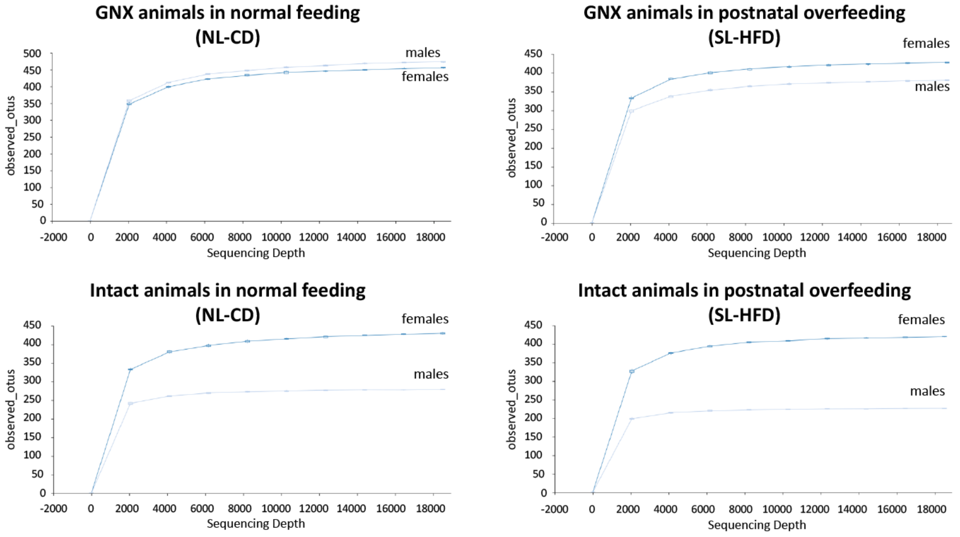

	Observed_otus	Shannon
GNX animals in normal feeding	0.495	0.834
GNX animals in postnatal overfeeding	0.294	0.753
Intact animals in normal feeding	0.027	0.016
Intact animals in postnatal overfeeding	0.021	0.027

Kruskal-Wallis statistical analysis p-values.

B **Gonadectomy effect on alpha diversity.**

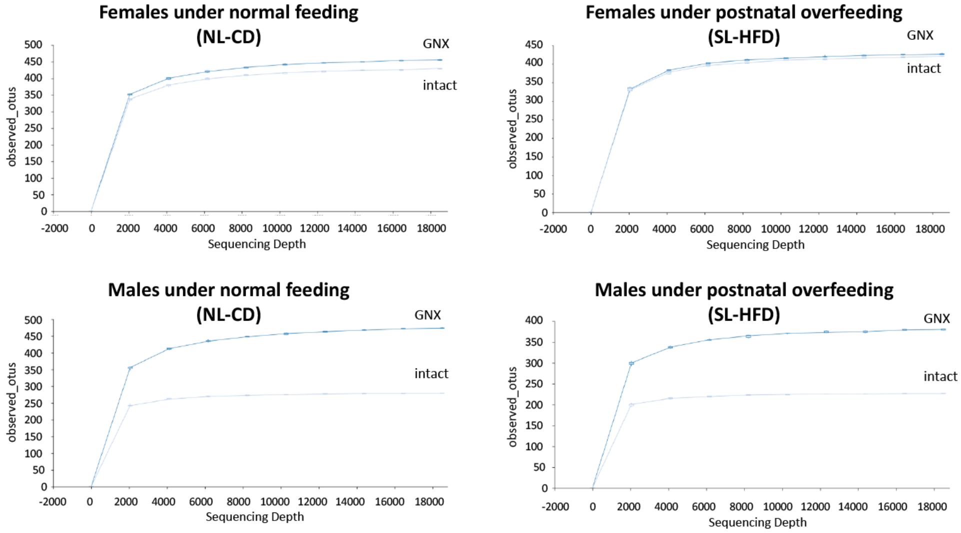

	Observed_otus	Shannon
Females under normal feeding	0.600	0.916
Females under postnatal overfeeding	1.000	0.208
Males under normal feeding	0.046	0.027
Males under postnatal overfeeding	0.059	0.074

Kruskal-Wallis statistical analysis p-values.

Supplementary Figure 1. Diversity indexes according to sex, gonadectomy and feeding conditions. (**A**) Sex effect on alpha diversity; (**B**) Gonadectomy effect on alpha diversity.

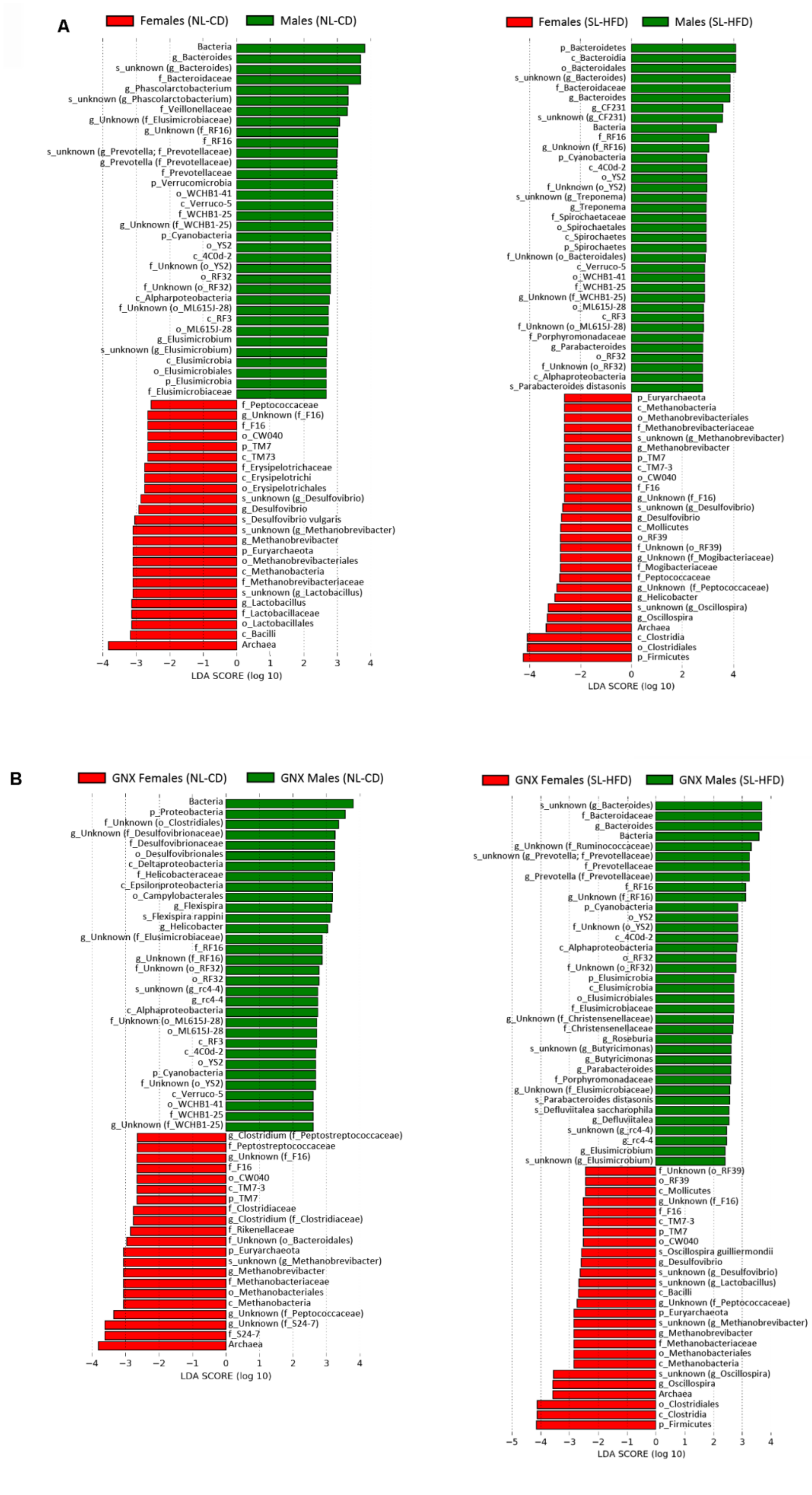

Supplementary Figure 2. Linear discriminant analysis (LDA) scores between sexes under normal feeding and overfeeding conditions, in intact (**A**) and gonadectomized (**B**) animals.

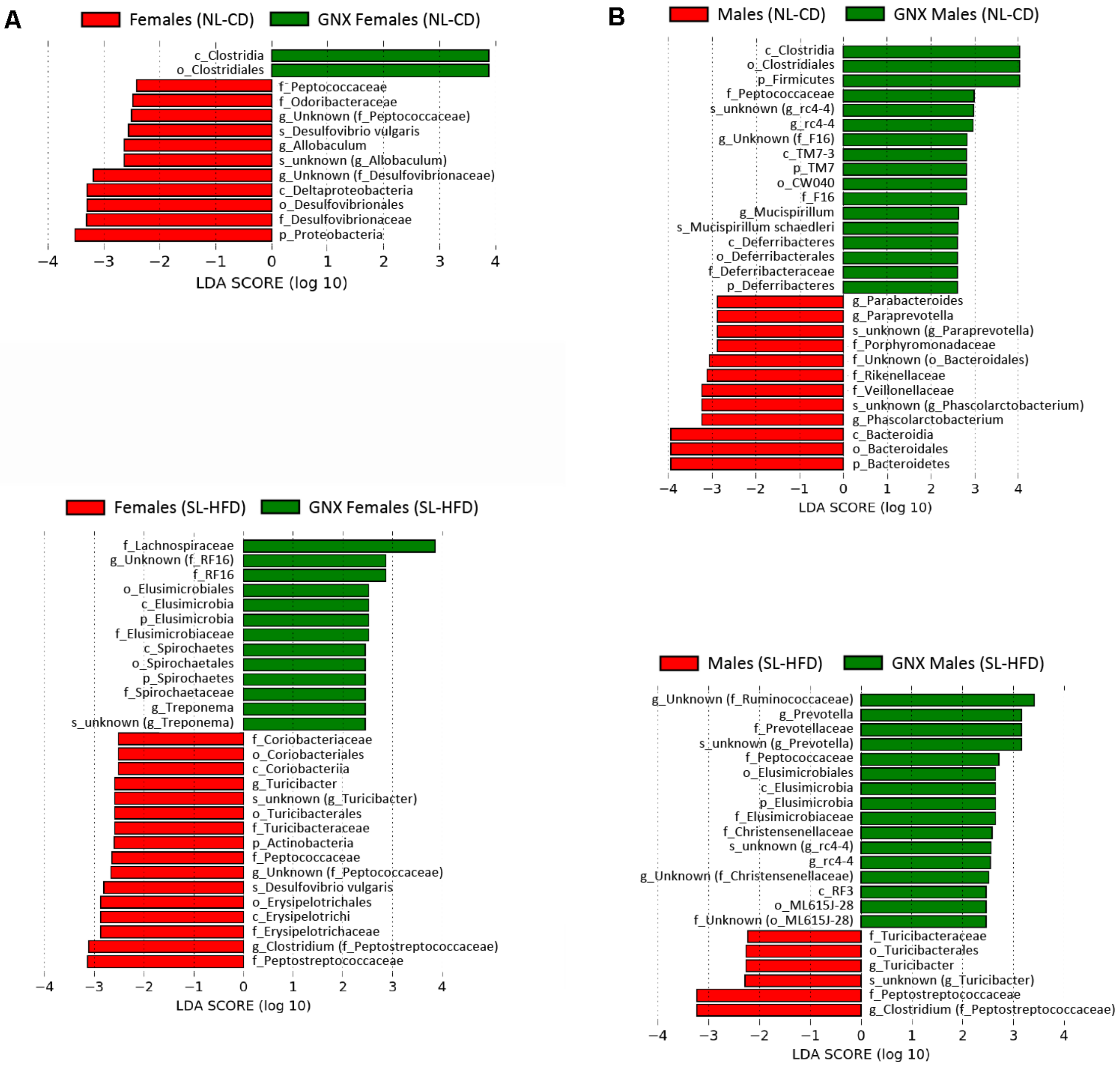

Supplementary Figure 3. Linear discriminant analysis (LDA) scores between intact and gonadectomized animals, under normal feeding and overfeeding conditions in females (**A**) and males (**B**).
