# Supplementary material for: Interplay between gonadal hormones and postnatal overfeeding in defining sex-dependent differences in gut microbiota architecture
Source: Aging (Albany NY). 2020 Oct 27;12(20):19979–20000. doi: 10.18632/aging.104140 (PMC7655199; doi:10.18632/aging.104140)
Supplement: Supplementary Table 1 [file aging-12-104140-s002..pdf]

SUPPLEMENTARY TABLE

Supplementary Table 1. Bacterial genera with different abundance between sexes in intact and gonadectomized animals.

	Normal feeding		*Postnatal overfeeding*	
	non-GNX	***GNX***	***non-GNX***	***GNX***
Methanobrevibacter	F	F	F	F
Bacteroides	M		M	M
Parabacteroides			M	M
Prevotella (Prevotellaceae)	M			M
Unknown (f_RF16)	M	M	M	M
Unknown (f_S24-7)		F		
Butyricimonas				M
CF231			M	
Unknown (Elusimicrobiaceae)	M	M		M
Elusimicrobium	M			M
Lactobacillus	F			
Unknown (Christensenellaceae)				M
Clostridium (Clostridiaceae)		F		
Roseburia				M
Unknown (Peptococcaceae)		F	F	F
rc4-4		M		M
Clostridium (Peptostreptococcaceae)		F		
Unknown (Ruminococcaceae)				M
Oscillospira			F	F
Phascolarctobacterium	M			
Unknown (Mogibacteriaceae)			F	
Unknown (Desulfovibrionaceae)		M		
Desulfovibrio	F		F	F
Flexispira		M		
Helicobacter		M	F	
Treponema			M	
Unknown (f_F16)	F	F	F	F
Unknown (f_WCHB1-25)	M	M	M	
Defluviitalea				M

GNX: gonadectomized animals. Non-GNX: intact animals. M: higher abundance in males. F: higher abundance in females.
