# Supplementary material for: Interplay between gonadal hormones and postnatal overfeeding in defining sex-dependent differences in gut microbiota architecture
Source: Aging (Albany NY). 2020 Oct 27;12(20):19979–20000. doi: 10.18632/aging.104140 (PMC7655199; doi:10.18632/aging.104140)
Supplement: Supplementary Table 2 [file aging-12-104140-s003..docx]

**Supplementary Table 2. Relationship between the bacterial genera identified by LEfSe analysis and the expression levels of the miRNAs in large intestine.** Pearson’s correlation analysis coefficient (Corr.) and P-value.

|  |  | rno-miR-6329 | rno-miR-3120 | rno-miR-27a-3p | rno-miR-10a-3p | rno-miR-421-3p | rno-miR-9b-5p | rno-miR-382-5p | rno-miR-672-5p | rno-miR-483-5p | rno-miR-365-3p | rno-miR-187-3p | rno-miR-1843b-3p | rno-miR-191a-5p | rno-miR-448-5p | rno-miR-219a-5p | rno-miR-99b-5p | rno-let-7e-3p | rno-miR-21-5p | rno-miR-3084b-5p | rno-miR-369-3p | rno-miR-126a-3p |
| --- | --- | --- | --- | --- | --- | --- | --- | --- | --- | --- | --- | --- | --- | --- | --- | --- | --- | --- | --- | --- | --- | --- |
| *Methanobrevibacter* | Corr. | -0.129 | -0.408 | -0.587 | -0.006 | -0.658 | 0.268 | -0.043 | 0.127 | **0.912** | -0.432 | -0.245 | -0.089 | 0.838 | -0.451 | -0.158 | **0.956** | **0.931** | -0.495 | -0.520 | -0.488 | -0.519 |
|  | p-value | 0.782 | 0.363 | 0.126 | 0.990 | 0.108 | 0.560 | 0.927 | 0.786 | **0.004** | 0.332 | 0.597 | 0.850 | 0.019 | 0.309 | 0.735 | **0.001** | **0.002** | 0.212 | 0.232 | 0.220 | 0.232 |
| *Parabacteroides* | Corr. | -0.250 | 0.845 | 0.486 | -0.323 | 0.402 | -0.330 | -0.434 | -0.246 | -0.313 | **0.929** | 0.711 | 0.078 | -0.688 | 0.337 | -0.134 | -0.561 | -0.668 | 0.481 | **0.932** | 0.568 | 0.288 |
|  | p-value | 0.588 | 0.017 | 0.222 | 0.479 | 0.371 | 0.470 | 0.331 | 0.596 | 0.495 | **0.002** | 0.073 | 0.867 | 0.088 | 0.461 | 0.775 | 0.190 | 0.101 | 0.228 | **0.002** | 0.142 | 0.532 |
| *Unknown (f_RF16)* | Corr. | -0.230 | 0.398 | 0.750 | 0.381 | 0.836 | -0.085 | -0.299 | -0.721 | -0.615 | 0.305 | 0.170 | -0.250 | **-0.917** | 0.714 | 0.451 | -0.666 | -0.801 | 0.519 | 0.515 | 0.600 | 0.586 |
|  | p-value | 0.619 | 0.376 | 0.032 | 0.399 | 0.019 | 0.856 | 0.515 | 0.068 | 0.142 | 0.507 | 0.716 | 0.588 | **0.004** | 0.072 | 0.310 | 0.102 | 0.031 | 0.187 | 0.237 | 0.116 | 0.167 |
| *Butyricimonas* | Corr. | -0.200 | 0.456 | **0.921** | -0.075 | 0.863 | -0.300 | -0.448 | -0.384 | -0.584 | 0.634 | 0.058 | -0.039 | -0.681 | 0.496 | -0.041 | -0.565 | -0.543 | **0.925** | 0.576 | **0.935** | 0.867 |
|  | p-value | 0.667 | 0.304 | **0.001** | 0.874 | 0.012 | 0.513 | 0.313 | 0.395 | 0.168 | 0.126 | 0.901 | 0.934 | 0.092 | 0.257 | 0.930 | 0.186 | 0.208 | **0.001** | 0.176 | **0.001** | 0.011 |
| *CF231* | Corr. | -0.339 | **0.904** | 0.106 | -0.414 | -0.016 | -0.314 | -0.471 | -0.175 | 0.034 | **0.953** | **0.922** | 0.059 | -0.387 | 0.130 | -0.311 | -0.170 | -0.351 | 0.125 | **0.950** | 0.241 | -0.086 |
|  | p-value | 0.458 | **0.005** | 0.803 | 0.356 | 0.973 | 0.493 | 0.286 | 0.707 | 0.943 | **0.001** | **0.003** | 0.899 | 0.391 | 0.782 | 0.497 | 0.716 | 0.440 | 0.768 | **0.001** | 0.565 | 0.854 |
| *Paraprevotella* | Corr. | -0.464 | -0.605 | -0.361 | 0.007 | -0.386 | **0.946** | -0.342 | 0.245 | 0.310 | -0.644 | -0.361 | 0.448 | 0.449 | 0.434 | 0.640 | 0.488 | 0.621 | -0.320 | -0.721 | -0.225 | -0.301 |
|  | p-value | 0.294 | 0.150 | 0.380 | 0.988 | 0.392 | **0.001** | 0.453 | 0.597 | 0.498 | 0.119 | 0.426 | 0.313 | 0.312 | 0.331 | 0.121 | 0.267 | 0.137 | 0.439 | 0.068 | 0.592 | 0.511 |
| *Mucispirillum* | Corr. | **0.903** | -0.388 | -0.173 | 0.201 | -0.236 | -0.477 | **0.909** | -0.035 | 0.102 | -0.115 | -0.440 | -0.639 | 0.437 | -0.561 | -0.330 | 0.374 | 0.216 | -0.184 | -0.241 | -0.308 | -0.064 |
|  | p-value | **0.005** | 0.389 | 0.681 | 0.665 | 0.611 | 0.279 | **0.005** | 0.941 | 0.827 | 0.806 | 0.323 | 0.123 | 0.327 | 0.190 | 0.469 | 0.408 | 0.642 | 0.662 | 0.603 | 0.458 | 0.892 |
| *Unknown (Elusimicrobiaceae)* | Corr. | -0.161 | 0.332 | **0.982** | 0.117 | **0.968** | -0.303 | -0.357 | -0.692 | -0.773 | 0.188 | -0.153 | -0.105 | -0.685 | 0.365 | -0.066 | -0.588 | -0.518 | **0.922** | 0.440 | 0.888 | **0.952** |
|  | p-value | 0.761 | 0.467 | **0.000** | 0.825 | **0.001** | 0.560 | 0.487 | 0.128 | 0.072 | 0.722 | 0.773 | 0.842 | 0.090 | 0.477 | 0.902 | 0.219 | 0.292 | **0.003** | 0.383 | 0.008 | **0.003** |
| *Unknown (Peptococcaceae)* | Corr. | -0.018 | -0.600 | -0.395 | -0.588 | -0.356 | 0.638 | -0.006 | **0.928** | 0.036 | -0.343 | -0.153 | 0.896 | 0.817 | -0.340 | -0.204 | 0.023 | 0.393 | -0.091 | -0.580 | -0.149 | -0.325 |
|  | p-value | 0.972 | 0.208 | 0.381 | 0.165 | 0.434 | 0.173 | 0.991 | **0.003** | 0.946 | 0.506 | 0.743 | 0.016 | 0.047 | 0.510 | 0.698 | 0.965 | 0.440 | 0.845 | 0.227 | 0.750 | 0.529 |
| *Clostridium (Peptostreptococcaceae)* | Corr. | -0.318 | 0.812 | -0.271 | -0.644 | -0.281 | -0.070 | -0.341 | 0.522 | -0.120 | 0.671 | 0.812 | **0.901** | -0.232 | -0.031 | -0.443 | -0.245 | -0.180 | -0.107 | 0.569 | -0.079 | -0.381 |
|  | p-value | 0.486 | 0.026 | 0.516 | 0.118 | 0.542 | 0.881 | 0.454 | 0.230 | 0.798 | 0.099 | 0.026 | **0.006** | 0.616 | 0.948 | 0.320 | 0.596 | 0.700 | 0.800 | 0.183 | 0.853 | 0.399 |
| *Unknown (Ruminococcaceae)* | Corr. | -0.015 | -0.105 | 0.814 | 0.513 | **0.919** | -0.120 | -0.054 | -0.395 | -0.644 | -0.578 | -0.529 | -0.142 | -0.596 | 0.199 | 0.250 | -0.545 | -0.462 | 0.669 | -0.023 | 0.588 | 0.850 |
|  | p-value | 0.974 | 0.823 | 0.014 | 0.239 | **0.003** | 0.797 | 0.908 | 0.381 | 0.118 | 0.174 | 0.222 | 0.761 | 0.158 | 0.668 | 0.589 | 0.205 | 0.297 | 0.070 | 0.962 | 0.125 | 0.015 |
| *Phascolarctobacterium* | Corr. | -0.405 | 0.279 | 0.191 | 0.063 | 0.397 | 0.498 | -0.499 | -0.610 | -0.385 | 0.340 | 0.690 | 0.079 | -0.552 | **0.926** | 0.713 | -0.397 | -0.573 | 0.039 | 0.314 | 0.208 | 0.019 |
|  | p-value | 0.368 | 0.544 | 0.651 | 0.892 | 0.378 | 0.256 | 0.254 | 0.146 | 0.394 | 0.455 | 0.086 | 0.866 | 0.199 | **0.003** | 0.072 | 0.378 | 0.179 | 0.926 | 0.492 | 0.622 | 0.967 |
| *Treponema* | Corr. | -0.144 | -0.270 | 0.131 | **0.922** | 0.252 | 0.258 | 0.119 | -0.569 | -0.458 | -0.256 | -0.179 | -0.260 | -0.408 | 0.578 | **0.979** | -0.294 | -0.374 | -0.211 | -0.239 | -0.124 | -0.023 |
|  | p-value | 0.757 | 0.559 | 0.757 | **0.003** | 0.586 | 0.576 | 0.800 | 0.183 | 0.301 | 0.580 | 0.700 | 0.573 | 0.364 | 0.174 | **0.000** | 0.522 | 0.409 | 0.616 | 0.606 | 0.769 | 0.961 |
