# Supplementary material for: Interplay between gonadal hormones and postnatal overfeeding in defining sex-dependent differences in gut microbiota architecture
Source: Aging (Albany NY). 2020 Oct 27;12(20):19979–20000. doi: 10.18632/aging.104140 (PMC7655199; doi:10.18632/aging.104140)
Supplement: Supplementary Table 3 [file aging-12-104140-s004..docx]

**Supplementary Table 3. Relationship between the bacterial species identified by LEfSe analysis and the expression levels of the miRNAs in large intestine.** Pearson’s correlation analysis coefficient (Corr.) and P-value.

|  |  | rno-miR-6329 | rno-miR-3120 | rno-miR-3576 | rno-miR-27a-3p | rno-miR-10a-3p | rno-miR-421-3p | rno-miR-9b-5p | rno-miR-382-5p | rno-miR-672-5p | rno-miR-483-5p | rno-miR-365-3p | rno-miR-187-3p | rno-miR-505-5p | rno-miR-1843b-3p | rno-miR-191a-5p | rno-miR-448-5p | rno-miR-219a-5p | rno-miR-3590-3p | rno-miR-99b-5p | rno-let-7e-3p | rno-miR-21-5p | rno-miR-3084b-5p | rno-miR-369-3p | rno-miR-126a-3p |
| --- | --- | --- | --- | --- | --- | --- | --- | --- | --- | --- | --- | --- | --- | --- | --- | --- | --- | --- | --- | --- | --- | --- | --- | --- | --- |
| *Unknown (Methanobrevibacter)* | Corr. | -0.129 | -0.408 | 0.689 | -0.587 | -0.006 | -0.658 | 0.268 | -0.043 | 0.127 | **0.912** | -0.433 | -0.245 | 0.530 | -0.089 | 0.838 | -0.452 | -0.158 | 0.259 | **0.956** | **0.931** | -0.495 | -0.520 | -0.488 | -0.519 |
|  | p-value | 0.783 | 0.363 | 0.087 | 0.126 | 0.990 | 0.108 | 0.561 | 0.928 | 0.786 | **0.004** | 0.332 | 0.597 | 0.221 | 0.850 | 0.019 | 0.309 | 0.735 | 0.574 | **0.001** | **0.002** | 0.212 | 0.232 | 0.220 | 0.232 |
| *Unknown (Parabacteroides)* | Corr. | -0.250 | 0.855 | -0.087 | 0.148 | -0.592 | 0.058 | -0.279 | -0.408 | 0.069 | -0.115 | **0.916** | 0.853 | 0.063 | 0.504 | -0.421 | 0.127 | -0.368 | -0.437 | -0.357 | -0.421 | 0.254 | **0.932** | 0.330 | -0.025 |
|  | p-value | 0.589 | 0.014 | 0.854 | 0.727 | 0.162 | 0.902 | 0.545 | 0.364 | 0.883 | 0.807 | **0.004** | 0.015 | 0.893 | 0.248 | 0.347 | 0.787 | 0.417 | 0.327 | 0.432 | 0.346 | 0.544 | **0.002** | 0.425 | 0.957 |
| *Parabacteroides distasonis* | Corr. | -0.267 | 0.829 | -0.352 | 0.587 | -0.185 | 0.521 | -0.323 | -0.439 | -0.400 | -0.358 | 0.899 | 0.635 | -0.410 | -0.076 | -0.768 | 0.420 | -0.047 | -0.188 | -0.578 | -0.724 | 0.531 | **0.907** | 0.62 | 0.394 |
|  | p-value | 0.563 | 0.021 | 0.439 | 0.126 | 0.692 | 0.230 | 0.480 | 0.324 | 0.374 | 0.430 | 0.006 | 0.126 | 0.361 | 0.872 | 0.044 | 0.349 | 0.921 | 0.686 | 0.174 | 0.066 | 0.176 | **0.005** | 0.101 | 0.382 |
| *Unknown (Butyricimonas)* | Corr. | -0.200 | 0.456 | -0.273 | **0.921** | -0.074 | 0.863 | -0.300 | -0.448 | -0.384 | -0.585 | 0.634 | 0.058 | -0.292 | -0.039 | -0.681 | 0.496 | -0.041 | -0.310 | -0.565 | -0.543 | **0.925** | 0.575 | **0.935** | 0.867 |
|  | p-value | 0.667 | 0.304 | 0.553 | **0.001** | 0.874 | 0.012 | 0.513 | 0.313 | 0.395 | 0.168 | 0.126 | 0.901 | 0.525 | 0.934 | 0.092 | 0.257 | 0.930 | 0.499 | 0.186 | 0.208 | **0.001** | 0.177 | **0.001** | 0.011 |
| *Unknown (CF231)* | Corr. | -0.339 | **0.904** | -0.017 | 0.107 | -0.414 | -0.015 | -0.314 | -0.471 | -0.175 | 0.034 | **0.953** | **0.922** | -0.080 | 0.060 | -0.388 | 0.130 | -0.311 | -0.246 | -0.170 | -0.351 | 0.126 | **0.951** | 0.242 | -0.086 |
|  | p-value | 0.458 | **0.005** | 0.971 | 0.802 | 0.356 | 0.974 | 0.493 | 0.286 | 0.707 | 0.943 | **0.001** | **0.003** | 0.865 | 0.899 | 0.390 | 0.782 | 0.497 | 0.594 | 0.715 | 0.440 | 0.767 | **0.001** | 0.564 | 0.855 |
| *Unknown (Paraprevotella)* | Corr. | -0.464 | -0.605 | 0.568 | -0.361 | 0.007 | -0.386 | **0.946** | -0.342 | 0.245 | 0.310 | -0.644 | -0.362 | 0.306 | 0.448 | 0.449 | 0.433 | 0.640 | 0.682 | 0.488 | 0.621 | -0.320 | -0.721 | -0.225 | -0.301 |
|  | p-value | 0.294 | 0.150 | 0.184 | 0.380 | 0.988 | 0.392 | **0.001** | 0.453 | 0.596 | 0.498 | 0.118 | 0.426 | 0.504 | 0.313 | 0.312 | 0.332 | 0.122 | 0.091 | 0.267 | 0.136 | 0.439 | 0.068 | 0.592 | 0.511 |
| *Mucispirillum schaedleri* | Corr. | **0.904** | -0.389 | -0.200 | -0.172 | 0.201 | -0.235 | -0.478 | **0.909** | -0.033 | 0.101 | -0.116 | -0.441 | -0.157 | -0.638 | 0.435 | -0.560 | -0.329 | -0.281 | 0.370 | 0.213 | -0.183 | -0.241 | -0.307 | -0.063 |
|  | p-value | **0.005** | 0.389 | 0.667 | 0.683 | 0.665 | 0.612 | 0.278 | **0.005** | 0.943 | 0.830 | 0.805 | 0.322 | 0.737 | 0.123 | 0.329 | 0.191 | 0.471 | 0.542 | 0.414 | 0.646 | 0.664 | 0.602 | 0.460 | 0.893 |
| *Unknown (Lactobacillus)* | Corr. | -0.133 | -0.311 | **0.902** | -0.431 | -0.492 | -0.443 | 0.179 | -0.128 | 0.891 | 0.208 | -0.276 | -0.065 | **0.917** | 0.739 | 0.789 | -0.399 | -0.365 | -0.424 | 0.172 | 0.493 | -0.168 | -0.371 | -0.238 | -0.346 |
|  | p-value | 0.776 | 0.496 | **0.005** | 0.286 | 0.262 | 0.319 | 0.702 | 0.785 | 0.007 | 0.655 | 0.548 | 0.891 | **0.004** | 0.057 | 0.035 | 0.375 | 0.420 | 0.343 | 0.713 | 0.261 | 0.690 | 0.412 | 0.571 | 0.447 |
| *Ruminococcus flavefaciens* | Corr. | -0.100 | -0.427 | -0.383 | 0.068 | **0.953** | 0.330 | 0.481 | 0.236 | -0.585 | -0.394 | -0.454 | -0.446 | -0.704 | -0.222 | -0.253 | 0.561 | 0.877 | **0.911** | -0.142 | -0.249 | -0.219 | -0.428 | -0.186 | 0.070 |
|  | p-value | 0.832 | 0.339 | 0.396 | 0.874 | **0.001** | 0.469 | 0.275 | 0.611 | 0.168 | 0.382 | 0.306 | 0.316 | 0.077 | 0.632 | 0.583 | 0.190 | 0.009 | **0.004** | 0.762 | 0.590 | 0.602 | 0.338 | 0.660 | 0.882 |
| *Unknown (Phascolarctobacterium)* | Corr. | -0.405 | 0.279 | -0.280 | 0.191 | 0.065 | 0.398 | 0.498 | -0.499 | -0.610 | -0.386 | 0.340 | 0.689 | -0.607 | 0.079 | -0.553 | **0.926** | 0.714 | 0.638 | -0.398 | -0.573 | 0.040 | 0.314 | 0.208 | 0.020 |
|  | p-value | 0.368 | 0.545 | 0.543 | 0.650 | 0.890 | 0.376 | 0.256 | 0.254 | 0.145 | 0.392 | 0.456 | 0.087 | 0.148 | 0.866 | 0.198 | **0.003** | 0.072 | 0.123 | 0.377 | 0.179 | 0.925 | 0.493 | 0.621 | 0.966 |
| *Unknown (Treponema)* | Corr. | -0.144 | -0.270 | -0.300 | 0.131 | **0.922** | 0.252 | 0.258 | 0.119 | -0.569 | -0.458 | -0.256 | -0.180 | -0.690 | -0.260 | -0.408 | 0.578 | **0.979** | 0.724 | -0.294 | -0.373 | -0.211 | -0.239 | -0.124 | -0.023 |
|  | p-value | 0.758 | 0.558 | 0.513 | 0.757 | **0.003** | 0.585 | 0.576 | 0.800 | 0.183 | 0.301 | 0.580 | 0.700 | 0.086 | 0.573 | 0.364 | 0.174 | **<0.001** | 0.066 | 0.522 | 0.409 | 0.616 | 0.605 | 0.769 | 0.962 |
