# Supplementary material for: Interplay between gonadal hormones and postnatal overfeeding in defining sex-dependent differences in gut microbiota architecture
Source: Aging (Albany NY). 2020 Oct 27;12(20):19979–20000. doi: 10.18632/aging.104140 (PMC7655199; doi:10.18632/aging.104140)
Supplement: Supplementary Table 4 [file aging-12-104140-s005..docx]

**Supplementary Table 4. Relationship between the bacterial genera identified by LEfSe analysis and the expression levels of the miRNAs in small intestine.** Pearson’s correlation analysis coefficient (Corr.) and P-value.

|  |  | rno-miR-181a-5p | rno-miR-6330 | rno-miR-125a-3p | rno-miR-1912-5p | rno-miR-29a-5p | rno-miR-3561-3p | rno-miR-133a-3p | rno-miR-1843b-5p | rno-miR-1843a-5p | rno-miR-344b-3p | rno-miR-764-3p | rno-miR-759 | rno-miR-128-1-5p | rno-miR-186-5p | rno-miR-99b-3p | rno-miR-6322 | rno-miR-211-5p | rno-miR-449a-5p | rno-miR-760-3p | rno-let-7f-2-3p | rno-miR-497-3p | rno-miR-3553 | rno-miR-1249 | rno-miR-1956-5p |
| --- | --- | --- | --- | --- | --- | --- | --- | --- | --- | --- | --- | --- | --- | --- | --- | --- | --- | --- | --- | --- | --- | --- | --- | --- | --- |
| *Bacteroides* | Corr. | 0.690 | -0.277 | **0.917** | 0.592 | 0.247 | -0.047 | 0.656 | 0.851 | -0.446 | -0.287 | -0.449 | 0.409 | 0.59 | -0.393 | 0.610 | 0.809 | -0.159 | -0.044 | 0.584 | 0.623 | 0.250 | 0.530 | 0.548 | 0.437 |
|  | p-value | 0.086 | 0.547 | **0.004** | 0.122 | 0.593 | 0.920 | 0.109 | 0.015 | 0.268 | 0.533 | 0.265 | 0.363 | 0.123 | 0.383 | 0.108 | 0.015 | 0.734 | 0.925 | 0.169 | 0.135 | 0.589 | 0.221 | 0.203 | 0.279 |
| *Parabacteroides* | Corr. | **0.926** | -0.334 | 0.705 | 0.261 | -0.121 | -0.130 | 0.813 | **0.980** | -0.385 | -0.360 | -0.483 | 0.479 | 0.718 | -0.460 | 0.783 | **0.956** | -0.263 | -0.133 | 0.851 | 0.523 | 0.489 | 0.713 | 0.452 | 0.659 |
|  | p-value | **0.003** | 0.465 | 0.077 | 0.533 | 0.797 | 0.781 | 0.026 | **<0.001** | 0.347 | 0.428 | 0.225 | 0.277 | 0.045 | 0.299 | 0.022 | **<0.001** | 0.569 | 0.777 | 0.015 | 0.228 | 0.266 | 0.072 | 0.309 | 0.075 |
| *Prevotella* | Corr. | 0.291 | -0.107 | 0.818 | 0.481 | 0.035 | -0.273 | 0.056 | **0.929** | -0.133 | -0.211 | -0.267 | 0.862 | 0.166 | -0.438 | 0.151 | 0.485 | -0.221 | -0.017 | 0.391 | **0.943** | -0.183 | -0.043 | **0.940** | -0.006 |
|  | p-value | 0.526 | 0.820 | 0.025 | 0.227 | 0.941 | 0.554 | 0.905 | **0.002** | 0.753 | 0.649 | 0.522 | 0.013 | 0.695 | 0.325 | 0.721 | 0.223 | 0.635 | 0.971 | 0.386 | **0.001** | 0.694 | 0.927 | **0.002** | 0.989 |
| *Unknown (f_S24-7)* | Corr. | -0.090 | -0.504 | -0.486 | -0.199 | 0.201 | -0.122 | -0.032 | -0.351 | 0.570 | -0.494 | -0.334 | -0.513 | -0.300 | **0.930** | 0.008 | -0.079 | -0.259 | -0.593 | -0.496 | -0.438 | 0.205 | -0.030 | -0.446 | 0.064 |
|  | p-value | 0.848 | 0.249 | 0.268 | 0.636 | 0.665 | 0.795 | 0.946 | 0.440 | 0.140 | 0.260 | 0.418 | 0.239 | 0.470 | **0.002** | 0.985 | 0.853 | 0.574 | 0.161 | 0.258 | 0.326 | 0.659 | 0.949 | 0.316 | 0.881 |
| *Butyricimonas* | Corr. | 0.797 | -0.389 | 0.670 | 0.349 | -0.070 | -0.199 | 0.234 | **0.966** | -0.177 | -0.436 | -0.494 | 0.874 | 0.330 | -0.349 | 0.241 | 0.590 | -0.155 | -0.240 | 0.535 | 0.867 | 0.000 | 0.020 | **0.919** | 0.032 |
|  | p-value | 0.032 | 0.389 | 0.099 | 0.397 | 0.882 | 0.669 | 0.614 | **<0.001** | 0.674 | 0.328 | 0.213 | 0.010 | 0.424 | 0.443 | 0.565 | 0.124 | 0.739 | 0.604 | 0.216 | 0.011 | 1.000 | 0.966 | **0.003** | 0.940 |
| *CF231* | Corr. | 0.878 | -0.274 | 0.488 | 0.031 | -0.298 | -0.129 | **0.912** | 0.761 | -0.181 | -0.336 | -0.398 | 0.196 | 0.727 | -0.281 | **0.948** | **0.948** | -0.367 | -0.137 | 0.833 | 0.217 | 0.631 | **0.904** | 0.086 | **0.924** |
|  | p-value | 0.009 | 0.552 | 0.266 | 0.942 | 0.517 | 0.782 | **0.004** | 0.047 | 0.668 | 0.462 | 0.329 | 0.674 | 0.041 | 0.541 | **<0.001** | **<0.001** | 0.418 | 0.770 | 0.020 | 0.641 | 0.129 | **0.005** | 0.855 | **0.001** |
| *Mucispirillum* | Corr. | -0.384 | **0.901** | -0.03 | -0.266 | -0.340 | 0.081 | -0.458 | -0.447 | -0.211 | **0.905** | **0.904** | -0.158 | -0.185 | -0.457 | -0.351 | -0.540 | 0.177 | **0.909** | -0.135 | -0.131 | -0.545 | -0.152 | -0.255 | -0.181 |
|  | p-value | 0.395 | **0.006** | 0.949 | 0.524 | 0.456 | 0.862 | 0.302 | 0.314 | 0.616 | **0.005** | **0.002** | 0.735 | 0.661 | 0.302 | 0.395 | 0.167 | 0.705 | **0.005** | 0.773 | 0.779 | 0.206 | 0.744 | 0.580 | 0.667 |
| *Unknown (Elusimicrobiaceae)* | Corr. | 0.227 | -0.352 | 0.466 | 0.426 | -0.102 | -0.101 | 0.045 | **0.911** | -0.070 | -0.394 | -0.437 | **0.937** | 0.226 | -0.339 | 0.066 | 0.384 | 0.010 | -0.274 | 0.435 | 0.851 | -0.153 | -0.170 | **0.958** | -0.154 |
|  | p-value | 0.665 | 0.493 | 0.352 | 0.341 | 0.847 | 0.849 | 0.933 | **0.011** | 0.881 | 0.439 | 0.327 | **0.006** | 0.626 | 0.511 | 0.888 | 0.395 | 0.985 | 0.600 | 0.388 | 0.032 | 0.773 | 0.747 | **0.003** | 0.742 |
| *Elusimicrobium* | Corr. | -0.131 | -0.348 | 0.569 | **0.966** | 0.713 | 0.322 | 0.096 | 0.572 | -0.031 | -0.373 | -0.484 | 0.328 | 0.132 | 0.064 | 0.086 | 0.228 | 0.309 | -0.258 | -0.096 | 0.634 | -0.407 | -0.114 | 0.756 | -0.167 |
|  | p-value | 0.780 | 0.444 | 0.182 | **<0.001** | 0.072 | 0.481 | 0.839 | 0.180 | 0.942 | 0.410 | 0.224 | 0.472 | 0.756 | 0.891 | 0.840 | 0.588 | 0.499 | 0.576 | 0.837 | 0.126 | 0.364 | 0.807 | 0.049 | 0.692 |
| *Clostridium (Peptostreptococcaceae)* | Corr. | **0.911** | -0.253 | 0.178 | -0.452 | -0.361 | -0.229 | 0.832 | -0.055 | -0.301 | -0.214 | -0.261 | -0.149 | 0.487 | -0.226 | 0.641 | 0.676 | -0.362 | -0.212 | 0.716 | -0.187 | **0.970** | 0.732 | -0.270 | 0.721 |
|  | p-value | **0.004** | 0.585 | 0.703 | 0.261 | 0.427 | 0.622 | 0.020 | 0.906 | 0.469 | 0.645 | 0.532 | 0.750 | 0.221 | 0.627 | 0.086 | 0.066 | 0.425 | 0.647 | 0.070 | 0.688 | **<0.001** | 0.061 | 0.559 | 0.044 |
| *Unknown (Desulfovibrionaceae)* | Corr. | 0.712 | 0.271 | 0.844 | 0.123 | 0.035 | 0.079 | 0.639 | 0.245 | **-0.918** | 0.217 | 0.035 | -0.027 | 0.625 | -0.595 | 0.401 | 0.485 | 0.027 | 0.437 | 0.582 | 0.193 | 0.271 | 0.866 | 0.054 | 0.336 |
|  | p-value | 0.073 | 0.557 | 0.017 | 0.772 | 0.941 | 0.866 | 0.122 | 0.597 | **0.001** | 0.640 | 0.934 | 0.954 | 0.098 | 0.159 | 0.325 | 0.223 | 0.954 | 0.327 | 0.170 | 0.678 | 0.557 | 0.012 | 0.908 | 0.416 |
| *Treponema* | Corr. | -0.338 | -0.212 | 0.438 | 0.837 | **0.966** | **0.914** | -0.015 | -0.045 | -0.095 | -0.174 | -0.249 | -0.259 | 0.125 | 0.353 | -0.129 | -0.261 | **0.910** | -0.128 | -0.501 | -0.201 | -0.603 | -0.216 | -0.022 | -0.364 |
|  | p-value | 0.458 | 0.649 | 0.325 | 0.010 | **<0.001** | **0.004** | 0.974 | 0.923 | 0.823 | 0.710 | 0.552 | 0.575 | 0.768 | 0.437 | 0.762 | 0.532 | **0.004** | 0.784 | 0.252 | 0.666 | 0.152 | 0.642 | 0.962 | 0.376 |
| *Unknown (f_WCHB1-25)* | Corr. | 0.499 | -0.338 | 0.860 | 0.578 | 0.147 | -0.131 | 0.246 | **0.956** | -0.205 | -0.391 | -0.483 | 0.790 | 0.327 | -0.334 | 0.263 | 0.586 | -0.111 | -0.180 | 0.438 | 0.881 | -0.080 | 0.043 | **0.914** | 0.037 |
|  | p-value | 0.254 | 0.458 | 0.013 | 0.133 | 0.752 | 0.779 | 0.595 | **0.001** | 0.625 | 0.386 | 0.226 | 0.034 | 0.429 | 0.465 | 0.530 | 0.127 | 0.813 | 0.700 | 0.326 | 0.009 | 0.865 | 0.927 | **0.004** | 0.931 |
