# Supplementary material for: Interplay between gonadal hormones and postnatal overfeeding in defining sex-dependent differences in gut microbiota architecture
Source: Aging (Albany NY). 2020 Oct 27;12(20):19979–20000. doi: 10.18632/aging.104140 (PMC7655199; doi:10.18632/aging.104140)
Supplement: Supplementary Table 5 [file aging-12-104140-s006..docx]

**Supplementary Table 5. Relationship between the bacterial species identified by LEfSe analysis and the expression levels of the miRNAs in small intestine.** Pearson’s correlation analysis coefficient (Corr.) and P-value.

|  |  | rno-miR-181a-5p | rno-miR-6330 | rno-miR-125a-3p | rno-miR-1912-5p | rno-miR-29a-5p | rno-miR-3561-3p | rno-miR-133a-3p | rno-miR-1843b-5p | rno-miR-1843a-5p | rno-miR-344b-3p | rno-miR-764-3p | rno-miR-759 | rno-miR-23b-5p | rno-miR-128-1-5p | rno-miR-186-5p | rno-miR-99b-3p | rno-miR-6322 | rno-miR-211-5p | rno-miR-139-5p | rno-miR-449a-5p | rno-miR-760-3p | rno-let-7f-2-3p | rno-miR-497-3p | rno-miR-3553 | rno-miR-1249 | rno-miR-1956-5p |
| --- | --- | --- | --- | --- | --- | --- | --- | --- | --- | --- | --- | --- | --- | --- | --- | --- | --- | --- | --- | --- | --- | --- | --- | --- | --- | --- | --- |
| *Unknown (Bacteroides)* | Corr. | 0.684 | -0.273 | **0.919** | 0.596 | 0.249 | -0.048 | 0.651 | 0.849 | -0.442 | -0.285 | -0.446 | 0.410 | 0.559 | 0.587 | -0.392 | 0.608 | 0.807 | -0.160 | -0.040 | -0.041 | 0.579 | 0.626 | 0.241 | 0.527 | 0.550 | 0.435 |
|  | p-value | 0.090 | 0.553 | **0.003** | 0.119 | 0.591 | 0.919 | 0.113 | 0.016 | 0.273 | 0.536 | 0.268 | 0.361 | 0.192 | 0.126 | 0.385 | 0.110 | 0.016 | 0.732 | 0.932 | 0.931 | 0.173 | 0.132 | 0.602 | 0.224 | 0.200 | 0.281 |
| *Unknown (Parabacteroides)* | Corr. | **0.970** | -0.233 | 0.483 | -0.106 | -0.328 | -0.291 | **0.927** | 0.728 | -0.361 | -0.273 | -0.343 | 0.252 | **0.906** | 0.650 | -0.452 | 0.824 | **0.948** | -0.457 | -0.498 | -0.097 | **0.931** | 0.290 | 0.754 | 0.832 | 0.156 | 0.824 |
|  | p-value | **<0.001** | 0.616 | 0.272 | 0.802 | 0.473 | 0.527 | **0.003** | 0.064 | 0.379 | 0.553 | 0.406 | 0.585 | **0.005** | 0.081 | 0.309 | 0.012 | **<0.001** | 0.303 | 0.256 | 0.836 | **0.002** | 0.528 | 0.050 | 0.020 | 0.739 | 0.012 |
| *Parabacteroides distasonis* | Corr. | 0.838 | -0.357 | 0.745 | 0.409 | -0.050 | -0.059 | 0.735 | **0.962** | -0.311 | -0.396 | -0.526 | 0.557 | 0.656 | 0.704 | -0.428 | 0.752 | **0.920** | -0.182 | -0.206 | -0.167 | 0.786 | 0.586 | 0.364 | 0.628 | 0.547 | 0.590 |
|  | p-value | 0.019 | 0.432 | 0.055 | 0.315 | 0.915 | 0.899 | 0.060 | **0.001** | 0.453 | 0.379 | 0.180 | 0.194 | 0.109 | 0.051 | 0.338 | 0.031 | **0.001** | 0.696 | 0.657 | 0.720 | 0.036 | 0.167 | 0.422 | 0.131 | 0.204 | 0.124 |
| *Unknown (Prevotella, Prevotellaceae)* | Corr. | 0.301 | -0.115 | 0.823 | 0.487 | 0.039 | -0.268 | 0.064 | **0.931** | -0.134 | -0.219 | -0.276 | 0.861 | 0.057 | 0.172 | -0.434 | 0.158 | 0.490 | -0.218 | -0.055 | -0.023 | 0.393 | **0.942** | -0.181 | -0.038 | **0.940** | -0.002 |
|  | p-value | 0.512 | 0.806 | 0.023 | 0.221 | 0.933 | 0.560 | 0.892 | **0.002** | 0.752 | 0.637 | 0.508 | 0.013 | 0.904 | 0.684 | 0.331 | 0.709 | 0.217 | 0.638 | 0.907 | 0.961 | 0.383 | **0.002** | 0.698 | 0.935 | **0.002** | 0.996 |
| *Unknown (Butyricimonas)* | Corr. | 0.796 | -0.389 | 0.670 | 0.349 | -0.070 | -0.199 | 0.234 | **0.966** | -0.177 | -0.436 | -0.494 | 0.874 | 0.326 | 0.330 | -0.349 | 0.241 | 0.590 | -0.155 | -0.234 | -0.240 | 0.534 | 0.867 | -0.001 | 0.020 | **0.919** | 0.032 |
|  | p-value | 0.032 | 0.389 | 0.099 | 0.397 | 0.882 | 0.669 | 0.614 | **<0.001** | 0.674 | 0.328 | 0.214 | 0.010 | 0.475 | 0.424 | 0.442 | 0.566 | 0.124 | 0.740 | 0.614 | 0.605 | 0.217 | 0.011 | 0.999 | 0.967 | **0.003** | 0.941 |
| *Unknown (CF231)* | Corr. | 0.878 | -0.274 | 0.488 | 0.031 | -0.298 | -0.130 | **0.912** | 0.761 | -0.181 | -0.336 | -0.398 | 0.197 | 0.790 | 0.727 | -0.281 | **0.948** | **0.948** | -0.367 | -0.333 | -0.137 | 0.833 | 0.217 | 0.631 | **0.904** | 0.086 | **0.924** |
|  | p-value | 0.009 | 0.552 | 0.267 | 0.942 | 0.516 | 0.782 | **0.004** | 0.047 | 0.669 | 0.462 | 0.329 | 0.672 | 0.034 | 0.041 | 0.541 | **<0.001** | **<0.001** | 0.418 | 0.465 | 0.769 | 0.020 | 0.64 | 0.129 | **0.005** | 0.854 | **0.001** |
| *Mucispirillum schaedleri* | Corr. | -0.383 | **0.900** | -0.028 | -0.266 | -0.339 | 0.082 | -0.458 | -0.446 | -0.214 | **0.905** | **0.904** | -0.158 | -0.422 | -0.185 | -0.458 | -0.352 | -0.541 | 0.178 | -0.336 | **0.910** | -0.135 | -0.131 | -0.545 | -0.153 | -0.255 | -0.183 |
|  | p-value | 0.396 | **0.006** | 0.952 | 0.525 | 0.457 | 0.861 | 0.302 | 0.316 | 0.611 | **0.005** | **0.002** | 0.735 | 0.346 | 0.662 | 0.301 | 0.393 | 0.167 | 0.703 | 0.462 | **0.004** | 0.773 | 0.780 | 0.206 | 0.743 | 0.581 | 0.665 |
| *Unknown (Elusimicrobium)* | Corr. | -0.131 | -0.349 | 0.569 | **0.966** | 0.713 | 0.322 | 0.096 | 0.572 | -0.031 | -0.373 | -0.484 | 0.328 | -0.078 | 0.132 | 0.064 | 0.086 | 0.228 | 0.310 | 0.606 | -0.258 | -0.096 | 0.634 | -0.408 | -0.114 | 0.756 | -0.167 |
|  | p-value | 0.780 | 0.444 | 0.182 | **<0.001** | 0.072 | 0.481 | 0.838 | 0.180 | 0.941 | 0.410 | 0.224 | 0.472 | 0.869 | 0.755 | 0.891 | 0.840 | 0.588 | 0.499 | 0.149 | 0.576 | 0.838 | 0.126 | 0.364 | 0.807 | 0.049 | 0.692 |
| *Ruminococcus flavefaciens* | Corr. | -0.593 | -0.120 | 0.155 | 0.780 | 0.839 | 0.720 | -0.316 | -0.051 | 0.193 | -0.095 | -0.152 | -0.140 | -0.554 | -0.176 | 0.339 | -0.294 | -0.400 | 0.791 | **0.940** | -0.195 | -0.622 | -0.201 | -0.638 | -0.383 | 0.031 | -0.456 |
|  | p-value | 0.160 | 0.797 | 0.741 | 0.022 | 0.018 | 0.068 | 0.490 | 0.914 | 0.647 | 0.839 | 0.720 | 0.765 | 0.197 | 0.677 | 0.457 | 0.480 | 0.326 | 0.034 | **0.002** | 0.675 | 0.136 | 0.666 | 0.123 | 0.396 | 0.948 | 0.256 |
| *Unknown (Treponema)* | Corr. | -0.338 | -0.212 | 0.438 | 0.837 | **0.966** | **0.913** | -0.015 | -0.045 | -0.095 | -0.173 | -0.249 | -0.259 | -0.179 | 0.125 | 0.353 | -0.129 | -0.262 | **0.910** | 0.761 | -0.128 | -0.501 | -0.200 | -0.603 | -0.216 | -0.022 | -0.364 |
|  | p-value | 0.458 | 0.649 | 0.325 | 0.010 | **<0.001** | **0.004** | 0.974 | 0.923 | 0.823 | 0.710 | 0.553 | 0.575 | 0.701 | 0.768 | 0.437 | 0.761 | 0.531 | **0.004** | 0.047 | 0.785 | 0.252 | 0.666 | 0.152 | 0.642 | 0.962 | 0.376 |
