# Supplementary material for: Interplay between gonadal hormones and postnatal overfeeding in defining sex-dependent differences in gut microbiota architecture
Source: Aging (Albany NY). 2020 Oct 27;12(20):19979–20000. doi: 10.18632/aging.104140 (PMC7655199; doi:10.18632/aging.104140)
Supplement: Supplementary Table 6 [file aging-12-104140-s007..docx]

**Supplementary Table 6. KEGG pathways related with miRNAs expression in the intestine.**

| **KEGG pathway** | **P-value** |
| --- | --- |
| **1. Metabolism** |  |
| **1.0 Global and overview maps** |  |
| 2-Oxocarboxylic acid metabolism | 0.011 |
| Fatty acid metabolism | 0.009 |
| **1.3 Lipid metabolism** |  |
| Fatty acid biosynthesis | 0.001 |
| Fatty acid elongation | 0.028 |
| **1.5 Amino acid metabolism** |  |
| Lysine degradation | <0.001 |
| **1.7 Glycan biosynthesis and metabolism** |  |
| Other types of O-glycan biosynthesis | 0.003 |
| **1.8 Metabolism of cofactors and vitamins** |  |
| Vitamin B6 metabolism | 0.011 |
| **2. Genetic Information Processing** |  |
| **2.1 Transcription** |  |
| Spliceosome | 0.002 |
| **2.2 Translation** |  |
| mRNA surveillance pathway | 0.006 |
| **2.3 Folding, sorting and degradation** |  |
| Protein processing in endoplasmic reticulum | <0.001 |
| Ubiquitin mediated proteolysis | 0.025 |
| RNA degradation | 0.012 |
| **3. Environmental Information Processing** |  |
| **3.2 Signal transduction** |  |
| Rap1 signaling pathway | 0.011 |
| MAPK signaling pathway | <0.001 |
| ErbB signaling pathway | 0.018 |
| TGF-beta signaling pathway | <0.001 |
| Hippo signaling pathway | <0.001 |
| VEGF signaling pathway | 0.029 |
| TNF signaling pathway | 0.002 |
| HIF-1 signaling pathway | 0.006 |
| FoxO signaling pathway | <0.001 |
| Sphingolipid signaling pathway | <0.001 |
| AMPK signaling pathway | 0.002 |
| mTOR signaling pathway | 0.005 |
| **3.3 Signaling molecules and interaction** |  |
| ECM-receptor interaction | 0.005 |
| **4. Cellular Processes** |  |
| **4.1 Transport and catabolism** |  |
| Endocytosis | 0.001 |
| **4.2 Cell growth and death** |  |
| Cell cycle | <0.001 |
| Oocyte meiosis | <0.001 |
| p53 signaling pathway | 0.001 |
| **4.3 Cellular community - eukaryotes** |  |
| Focal adhesion | <0.001 |
| Adherens junction | <0.001 |
| Signaling pathways regulating pluripotency of stem cells | <0.001 |
| **5. Organismal Systems** |  |
| **5.1 Immune system** |  |
| Platelet activation | 0.030 |
| Fc gamma R-mediated phagocytosis | 0.005 |
| **5.2 Endocrine system** |  |
| Insulin signaling pathway | 0.001 |
| GnRH signaling pathway | 0.012 |
| Estrogen signaling pathway | 0.001 |
| Progesterone-mediated oocyte maturation | 0.001 |
| Prolactin signaling pathway | 0.030 |
| Thyroid hormone signaling pathway | <0.001 |
| **5.6 Nervous system** |  |
| Neurotrophin signaling pathway | <0.001 |
| **5.8 Development** |  |
| Axon guidance | <0.001 |
| **5.10 Environmental adaptation** |  |
| Circadian rhythm | 0.013 |
| **6. Human Diseases** |  |
| **6.1 Cancers: Overview** |  |
| Pathways in cancer | <0.001 |
| Central carbon metabolism in cancer | 0.005 |
| Choline metabolism in cancer | 0.006 |
| Transcriptional misregulation in cancer | <0.001 |
| Proteoglycans in cancer | <0.001 |
| Viral carcinogenesis | <0.001 |
| **6.2 Cancers: Specific types** |  |
| Colorectal cancer | <0.001 |
| Pancreatic cancer | <0.001 |
| Glioma | <0.001 |
| Thyroid cancer | <0.001 |
| Acute myeloid leukemia | 0.007 |
| Chronic myeloid leukemia | <0.001 |
| Melanoma | 0.007 |
| Renal cell carcinoma | <0.001 |
| Bladder cancer | <0.001 |
| Prostate cancer | <0.001 |
| Endometrial cancer | <0.001 |
| Small cell lung cancer | 0.012 |
| Non-small cell lung cancer | <0.001 |
| **6.4 Neurodegenerative diseases** |  |
| Prion diseases | <0.001 |
| **6.8 Infectious diseases: Bacterial** |  |
| Epithelial cell signaling in Helicobacter pylori infection | 0.047 |
| Salmonella infection | 0.005 |
| Shigellosis | <0.001 |
| Bacterial invasion of epithelial cells | <0.001 |
| **6.9 Infectious diseases: Viral** |  |
| Human T-cell leukemia virus 1 infection | 0.032 |
| Hepatitis B | <0.001 |
| Hepatitis C | 0.032 |
| Epstein-Barr virus infection | 0.001 |
| **6.10 Infectious diseases: Parasitic** |  |
| Toxoplasmosis | 0.033 |
| Chagas disease (American trypanosomiasis) | 0.005 |
