# Supplementary material for: Interplay between gonadal hormones and postnatal overfeeding in defining sex-dependent differences in gut microbiota architecture
Source: Aging (Albany NY). 2020 Oct 27;12(20):19979–20000. doi: 10.18632/aging.104140 (PMC7655199; doi:10.18632/aging.104140)
Supplement: Supplementary Table 7 [file aging-12-104140-s008..docx]

**Supplementary Table 7. KEGG pathways associated with microbiome-related miRNAs.**

| **KEGG pathway** | **Human miRNA** |
| --- | --- |
| 2-Oxocarboxylic acid metabolism (hsa01210) | hsa-miR-505-5p-L |
| Acute myeloid leukemia (hsa05221) | hsa-miR-181a-5p-S |
| Adherens junction (hsa04520) | hsa-miR-27a-3p-L; hsa-miR-10a-3p-L; hsa-miR-99b-5p-L; hsa-miR-181a-5p-S;hsa-miR-125a-3p-S;hsa-miR-186-5p-S; hsa-miR-99b-3p-S; hsa-miR-139-5p-S |
| Adrenergic signaling in cardiomyocytes (hsa04261) | hsa-miR-181a-5p-S |
| Allograft rejection (hsa05330) | hsa-miR-382-5p-L |
| Alzheimer's disease (hsa05010) | hsa-let-7e-3p-L |
| Amoebiasis (hsa05146) | hsa-miR-382-5p-L |
| AMPK signaling pathway (hsa04152) | hsa-miR-27a-3p-L; hsa-miR-186-5p-S |
| Antigen processing and presentation (hsa04612) | hsa-miR-382-5p-L; hsa-miR-211-5p-S |
| Arrhythmogenic right ventricular cardiomyopathy (ARVC) (hsa05412) | hsa-miR-505-5p-L |
| Axon guidance (hsa04360) | hsa-miR-27a-3p-L; hsa-miR-505-5p-L; hsa-miR-99b-5p-L |
| Bacterial invasion of epithelial cells (hsa05100) | hsa-miR-27a-3p-L; hsa-miR-21-5p-L; hsa-miR-181a-5p-S; hsa-miR-99b-3p-S |
| Biosynthesis of unsaturated fatty acids (hsa01040) | hsa-miR-21-5p-L |
| Biotin metabolism (hsa00780) | hsa-miR-181a-5p-S |
| Bladder cancer (hsa05219) | hsa-miR-27a-3p-L; hsa-miR-181a-5p-S |
| Cell cycle (hsa04110) | hsa-miR-27a-3p-L; hsa-miR-21-5p-L; hsa-miR-186-5p-S; hsa-miR-99b-3p-S |
| Central carbon metabolism in cancer (hsa05230) | hsa-miR-27a-3p-L; hsa-miR-382-5p-L; hsa-miR-181a-5p-S; hsa-miR-125a-3p-S |
| Chronic myeloid leukemia (hsa05220) | hsa-miR-27a-3p-L; hsa-miR-181a-5p-S; hsa-miR-186-5p-S |
| Circadian entrainment (hsa04713) | hsa-miR-369-3p-L |
| Colorectal cancer (hsa05210) | hsa-miR-27a-3p-L; hsa-miR-10a-3p-L; hsa-miR-21-5p-L; hsa-miR-181a-5p-S; hsa-miR-125a-3p-S; hsa-miR-186-5p-S |
| Cytokine-cytokine receptor interaction (hsa04060) | hsa-miR-211-5p-S |
| Dorso-ventral axis formation (hsa04320) | hsa-miR-181a-5p-S |
| ECM-receptor interaction (hsa04512) | hsa-miR-27a-3p-L; hsa-miR-382-5p-L; hsa-miR-125a-3p-S; hsa-miR-23b-5p-S; hsa-miR-211-5p-S |
| Endometrial cancer (hsa05213) | hsa-miR-27a-3p-L; hsa-miR-21-5p-L; hsa-miR-181a-5p-S; hsa-miR-139-5p-S |
| Epithelial cell signaling in Helicobacter pylori infection (hsa05120) | hsa-miR-10a-3p-L |
| Epstein-Barr virus infection (hsa05169) | hsa-miR-186-5p-S |
| ErbB signaling pathway (hsa04012) | hsa-miR-27a-3p-L; hsa-miR-139-5p-S |
| Estrogen signaling pathway (hsa04915) | hsa-miR-181a-5p-S; hsa-miR-139-5p-S |
| Fatty acid biosynthesis (hsa00061) | hsa-miR-27a-3p-L |
| Fatty acid degradation (hsa00071) | hsa-miR-21-5p-L; hsa-miR-29a-5p-S |
| Fatty acid elongation (hsa00062) | hsa-miR-21-5p-L; hsa-miR-29a-5p-S |
| Fatty acid metabolism (hsa01212) | hsa-miR-27a-3p-L; hsa-miR-21-5p-L |
| Focal adhesion (hsa04510) | hsa-miR-27a-3p-L; hsa-miR-125a-3p-S; hsa-miR-186-5p-S |
| FoxO signaling pathway (hsa04068) | hsa-miR-27a-3p-L; hsa-miR-10a-3p-L; hsa-miR-21-5p-L; hsa-miR-181a-5p-S; hsa-miR-125a-3p-S |
| Glioma (hsa05214) | hsa-miR-27a-3p-L; hsa-miR-181a-5p-S; hsa-miR-139-5p-S |
| Glycerolipid metabolism (hsa00561) | hsa-let-7e-3p-L |
| Glycosphingolipid biosynthesis - ganglio series (hsa00604) | hsa-miR-187-3p-L |
| Glycosphingolipid biosynthesis - lacto and neolacto series (hsa00601) | hsa-miR-505-5p-L |
| Hepatitis B (hsa05161) | hsa-miR-27a-3p-L; hsa-miR-10a-3p-L; hsa-miR-21-5p-L; hsa-miR-181a-5p-S |
| HIF-1 signaling pathway (hsa04066) | hsa-miR-181a-5p-S |
| Hippo signaling pathway (hsa04390) | hsa-miR-27a-3p-L; hsa-miR-10a-3p-L; hsa-miR-21-5p-L; hsa-miR-181a-5p-S; hsa-miR-29a-5p-S; hsa-miR-99b-3p-S; hsa-miR-139-5p-S |
| Insulin signaling pathway (hsa04910) | hsa-miR-27a-3p-L |
| Leukocyte transendothelial migration (hsa04670) | hsa-miR-99b-5p-L |
| Long-term depression (hsa04730) | hsa-miR-99b-5p-L |
| Lysine degradation (hsa00310) | hsa-miR-27a-3p-L; hsa-miR-505-5p-L; hsa-miR-21-5p-L; hsa-miR-181a-5p-S; hsa-miR-125a-3p-S; hsa-miR-186-5p-S |
| Melanoma (hsa05218) | hsa-miR-27a-3p-L; hsa-miR-181a-5p-S |
| Metabolism of xenobiotics by cytochrome P450 (hsa00980) | hsa-miR-497-3p-S |
| Morphine addiction (hsa05032) | hsa-miR-369-3p-L |
| mRNA surveillance pathway (hsa03015) | hsa-miR-29a-5p-S |
| mTOR signaling pathway (hsa04150) | hsa-miR-27a-3p-L; hsa-miR-382-5p-L |
| Mucin type O-Glycan biosynthesis (hsa00512) | hsa-miR-27a-3p-L; hsa-miR-99b-5p-L; hsa-miR-29a-5p-S |
| Neurotrophin signaling pathway (hsa04722) | hsa-miR-27a-3p-L; hsa-miR-181a-5p-S; hsa-miR-139-5p-S |
| NF-kappa B signaling pathway (hsa04064) | hsa-miR-139-5p-S |
| Non-homologous end-joining (hsa03450) | hsa-miR-99b-3p-S |
| Non-small cell lung cancer (hsa05223) | hsa-miR-27a-3p-L; hsa-miR-181a-5p-S |
| One carbon pool by folate (hsa00670) | hsa-miR-125a-3p-S |
| Oocyte meiosis (hsa04114) | hsa-miR-27a-3p-L; hsa-miR-29a-5p-S |
| Other types of O-glycan biosynthesis (hsa00514) | hsa-miR-505-5p-L |
| p53 signaling pathway (hsa04115) | hsa-miR-27a-3p-L; hsa-miR-21-5p-L; hsa-miR-181a-5p-S; hsa-miR-186-5p-S; hsa-miR-497-3p-S |
| Pancreatic cancer (hsa05212) | hsa-miR-27a-3p-L; hsa-miR-181a-5p-S |
| Pantothenate and CoA biosynthesis (hsa00770) | hsa-miR-181a-5p-S |
| Parkinson's disease (hsa05012) | hsa-miR-23b-5p-S; hsa-miR-99b-3p-S |
| Pathways in cancer (hsa05200) | hsa-miR-27a-3p-L; hsa-miR-10a-3p-L; hsa-miR-21-5p-L; hsa-miR-181a-5p-S; hsa-miR-186-5p-S |
| Phosphatidylinositol signaling system (hsa04070) | hsa-miR-483-5p-L |
| PI3K-Akt signaling pathway (hsa04151) | hsa-miR-382-5p-L; hsa-miR-181a-5p-S |
| PPAR signaling pathway (hsa03320) | hsa-let-7e-3p-L |
| Prion diseases (hsa05020) | hsa-miR-27a-3p-L; hsa-miR-382-5p-L; hsa-miR-186-5p-S |
| Progesterone-mediated oocyte maturation (hsa04914) | hsa-miR-27a-3p-L; hsa-miR-186-5p-S |
| Prolactin signaling pathway (hsa04917) | hsa-miR-21-5p-L; hsa-miR-181a-5p-S |
| Prostate cancer (hsa05215) | hsa-miR-27a-3p-L; hsa-miR-181a-5p-S; hsa-miR-186-5p-S; hsa-miR-139-5p-S |
| Protein processing in endoplasmic reticulum (hsa04141) | hsa-miR-27a-3p-L; hsa-miR-483-5p-L; hsa-miR-181a-5p-S; hsa-miR-186-5p-S; hsa-miR-211-5p-S |
| Proteoglycans in cancer (hsa05205) | hsa-miR-27a-3p-L; hsa-miR-10a-3p-L; hsa-miR-21-5p-L; hsa-miR-181a-5p-S; hsa-miR-125a-3p-S; hsa-miR-29a-5p-S; hsa-miR-186-5p-S |
| Regulation of actin cytoskeleton (hsa04810) | hsa-miR-181a-5p-S; hsa-miR-186-5p-S |
| Renal cell carcinoma (hsa05211) | hsa-miR-27a-3p-L; hsa-miR-181a-5p-S |
| RNA degradation (hsa03018) | hsa-miR-187-3p-L; hsa-miR-181a-5p-S; hsa-miR-497-3p-S |
| Salmonella infection (hsa05132) | hsa-miR-505-5p-L |
| Serotonergic synapse (hsa04726) | hsa-miR-483-5p-L |
| Shigellosis (hsa05131) | hsa-miR-27a-3p-L; hsa-miR-10a-3p-L |
| Signaling pathways regulating pluripotency of stem cells (hsa04550) | hsa-miR-27a-3p-L; hsa-let-7e-3p-L; hsa-miR-181a-5p-S |
| Small cell lung cancer (hsa05222) | hsa-miR-181a-5p-S |
| Sphingolipid metabolism (hsa00600) | hsa-miR-21-5p-L; hsa-miR-186-5p-S |
| Sphingolipid signaling pathway (hsa04071) | hsa-miR-27a-3p-L; hsa-miR-181a-5p-S |
| Spliceosome (hsa03040) | hsa-miR-99b-5p-L |
| Steroid biosynthesis (hsa00100) | hsa-miR-23b-5p-S; hsa-miR-186-5p-S |
| Sulfur metabolism (hsa00920) | hsa-miR-505-5p-L |
| Synaptic vesicle cycle (hsa04721) | hsa-miR-187-3p-L |
| Terpenoid backbone biosynthesis (hsa00900) | hsa-miR-181a-5p-S |
| TGF-beta signaling pathway (hsa04350) | hsa-miR-27a-3p-L; hsa-miR-10a-3p-L; hsa-miR-125a-3p-S; hsa-miR-29a-5p-S; hsa-miR-186-5p-S |
| Thyroid cancer (hsa05216) | hsa-miR-27a-3p-L; hsa-miR-21-5p-L; hsa-miR-181a-5p-S; hsa-miR-125a-3p-S |
| Thyroid hormone signaling pathway (hsa04919) | hsa-miR-27a-3p-L; hsa-miR-21-5p-L |
| Transcriptional misregulation in cancer (hsa05202) | hsa-miR-27a-3p-L; hsa-miR-21-5p-L; hsa-miR-181a-5p-S; hsa-miR-186-5p-S |
| Tyrosine metabolism (hsa00350) | hsa-miR-369-3p-L |
| Ubiquitin mediated proteolysis (hsa04120) | hsa-miR-27a-3p-L |
| Valine, leucine and isoleucine biosynthesis (hsa00290) | hsa-miR-505-5p-L |
| Viral carcinogenesis (hsa05203) | hsa-miR-27a-3p-L; hsa-miR-181a-5p-S; hsa-miR-125a-3p-S; hsa-miR-186-5p-S |
| Viral myocarditis (hsa05416) | hsa-miR-382-5p-L |
| Vitamin B6 metabolism (hsa00750) | hsa-miR-186-5p-S |

hsa: *Homo sapiens*; -L: large intestine; -S: small intestine
